# The membrane skeleton is constitutively remodeled in neurons by calcium signaling

**DOI:** 10.1126/science.adn6712

**Published:** 2025-08-07

**Authors:** Evan Heller, Naina Kurup, Xiaowei Zhuang

**Affiliations:** 1 Howard Hughes Medical Institute, Harvard University, Cambridge, MA 02138, USA; 2 Department of Chemistry and Chemical Biology, Harvard University, Cambridge, MA 02138, USA; 3 Department of Physics, Harvard University, Cambridge, MA 02138, USA

## Abstract

The membrane skeleton in neurons adopts a unique periodic lattice structure, in which actin filaments, capped by adducin and tropomodulin, form ring-shaped structures connected by spectrin tetramers along neurites. This membrane-associated periodic skeleton (MPS) is important for many neuronal functions. Here, using live-cell super-resolution imaging, we found that the MPS is surprisingly dynamic, undergoing local disassembly and reformation constitutively in axons. MPS remodeling is driven by calcium signaling, leading to actin-ring destabilization through PKC-mediated adducin phosphorylation and to spectrin degradation by calpain. Formin, an actin-nucleating and -polymerizing enzyme, plays a dual role in MPS remodeling and maintenance. MPS remodeling is enhanced by neuronal activity and functionally facilitates endocytosis. Our results highlight the importance of a dynamic membrane skeletal structure in neuronal function.

The cytoskeleton plays an important role in a variety of cellular functions, ranging from mechanical support for cells and intracellular cargo transport, to mitosis of replicating cells and synaptic plasticity in neurons. In performing these functions, the cytoskeleton exhibits a fascinating dichotomy: it forms a rich and elaborate array of persistent structures out of filaments that are dynamic (1, 2). For example, actin filaments in the cell cortex form a network that confers mechanical support and maintains cell shape (3), while the network of intermediate filaments provides cells with additional strength and stiffness (4). Actin, intermediate filaments, and microtubules also help to shape and position intracellular organelles (4-5), and actin and microtubules additionally form molecular tracks for intracellular cargo transport by motor proteins (2). The stability of these cytoskeletal structures, however, is counterbalanced by dynamics, as even apparently long-lived networks are remodeled over time to meet the changing needs of the cell. This dichotomy between stability and plasticity is particularly important in the case of neurons, which must survive the entire lifespan of the animal and maintain long-term stability of neural networks (6), while simultaneously allowing local remodeling of neurites and synapses that enables learning and adaptation (7, 8).

In neurons, super-resolution imaging has revealed a quasi-one-dimensional, periodic submembrane cytoskeletal structure termed the membrane-associated periodic skeleton (MPS) (9). In this structure, actin filaments, capped by adducin and tropomodulin, form ring-shaped structures that are evenly spaced at ~180–190 nm intervals by spectrin tetramers along axons and some dendrites (9-15). The abundance of actin-capping proteins in the ring (9, 15) suggests that it may consist of multiple short, capped actin filaments, analogous to the short filaments present in the erythrocyte membrane skeleton, whereas an EM study suggests the possibility that the actin ring is made of a pair of long, intertwined filaments (16). The MPS is present in diverse neuronal cell types (17, 18) and is conserved across animal species (18). It plays a critical role in a variety of functions in neurons, including organizing membrane proteins (9, 19-23), signal transduction (23), mechanosensation (24), tension buffering (25), axon degeneration (26, 27), axon-diameter control (15, 28, 29), and neurite-neurite interactions (15, 22). Genetic perturbation of the MPS in mice causes a range of neurological impairments (30, 31), and hereditary mutations of MPS components in humans are associated with neurological diseases (32, 33).

Unlike other cytoskeletal structures, the dynamic properties of the MPS remain largely unexplored. In some specific functions, the MPS is actively degraded in response to an external stimulus. For example, when functioning as a signaling platform to enable G-protein coupled receptor (GPCR)- or cell-adhesion molecule (CAM)-mediated signal transduction in neurons, the MPS is actively degraded by calpain upon stimulation of GPCRs and CAMs, and such degradation modulates signaling strength (23). The MPS is also degraded upon trophic-deprivation-induced axon degeneration (26, 27). In addition, F-actin can be remodeled from actin rings in the MPS into longitudinal fibers in dendrites in response to synaptic activity stimulation (34). Neuronal activity stimulation can also cause an increase in longitudinal actin fibers in axons, with a modest reduction in actin ring periodicity, in the axon initial segment (35). Although these observations are limited to specific circumstances involving the application of strong external stimuli, they suggest the MPS has a capacity to remodel.

However, the MPS is generally considered to be a stable structure. The MPS is thought to provide the mechanical support required to maintain axon integrity during animal movement (9, 36). The role of the MPS as a scaffold for proper localization of functionally important membrane proteins (9, 19-23) similarly suggests a need for stability. Experimental evidence to date also suggests MPS stability: previous FRAP experiments have shown slow turnover of MPS components (10, 37, 38), and the MPS exhibits a high resistance to actin depolymerizing drugs (9, 10, 39). Due to the challenges associated with visualizing the MPS in live neurons, live-cell MPS imaging studies are relatively rare (10, 28, 40), and real-time live imaging to date has only revealed radial expansion and contraction of the MPS to accommodate large cargoes passing through axons (28). The dynamics of MPS therefore remain largely unknown.

## The MPS is dynamic and undergoes constitutive disassembly and reassembly

**Fig. 1.**
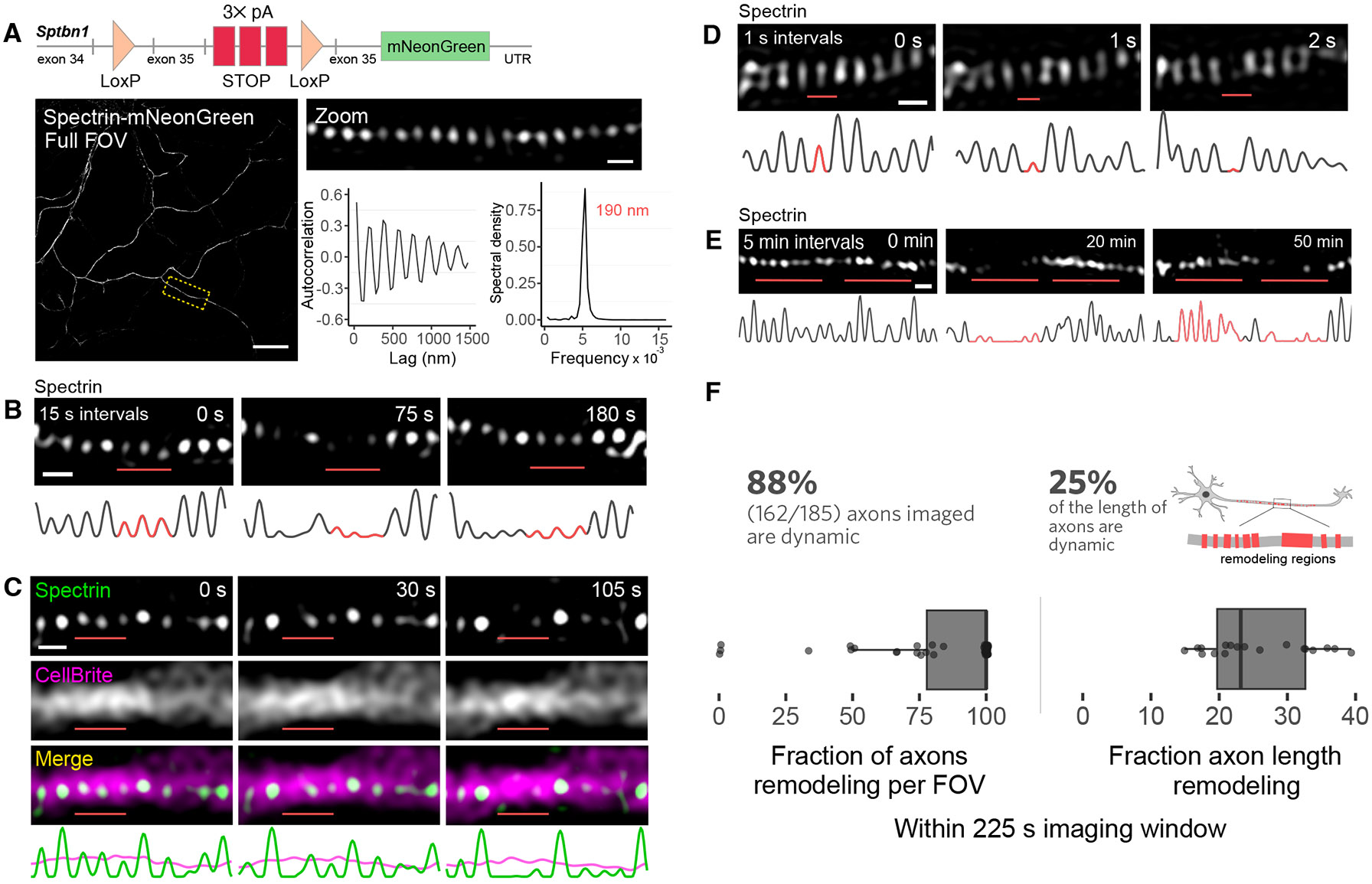
The MPS undergoes constitutive remodeling. (**A**) Characterization of MPS dynamics in the βII-spectrin mNeonGreen conditional knock-in mouse by live-cell SIM imaging. *Top*, schematic of the conditional knock-in construct, which expresses a βII-spectrin-mNeonGreen fusion protein in the presence of *Cre*. *Bottom*, SIM image of a DIV 15 neuron expressing specrin-mNeonGreen. Left: full field-of-view (FOV). Right top: Zoom-in of boxed region on the left. Right bottom: Autocorrelation function of the spectrin-mNeonGreen signal along the axons (left, averaged over 45 axonal region) and spectral density analysis of the autocorrelation function (right) showing a periodic distribution of βII-spectrin with ~190-nm spacing along the axon. (**B**) Example MPS remodeling (disassembly and reassembly) from live-cell SIM imaging at a 15-sec time resolution over a 225-sec imaging duration, with intensity traces shown below. Remodeling regions are highlighted in red. (**C**) Live-cell two-color SIM imaging of the MPS (top) and the membrane dye CellBrite Steady (middle), with composite images and intensity traces (green, βII-spectrin; magenta, CellBrite) shown below. (**D, E**) As in (B), but for MPS remodeling events from SIM images acquired at 1-sec (D) and 5-min (E) time resolution. Full sequence of the images taken at 5-min resolution is shown in fig. S3A. (**F**) *Left*, fraction of axons per FOV exhibiting MPS remodeling during a standardized 225-sec imaging window. *Right*, fraction of axon length undergoing MPS remodeling during the 225-sec imaging window. In all figures, elements in statistical quantification plots, including boxplot elements and error bars, are defined in the Statistics section of Materials and Methods. Statistics for (F) are shown in Table S2. Scale bars, 10 *μ*m (A), 250 nm (A, zoom; B–E).

To study the dynamic properties of the MPS in live neurons, we generated a Cre-inducible knock-in of mNeonGreen in the C-terminus of mouse βII-spectrin (spectrin-mNeonGreen, Fig. 1A) and used it to image the MPS in cultured hippocampal neurons at DIV (days in vitro) 14 – 16 using lattice structured illumination microscopy (SIM) (41). We observed that the MPS was highly dynamic and exhibited repeated cycles of disassembly and re-assembly even under unstimulated conditions (Fig. 1, B to E and Movie S1).

We performed SIM imaging across several different timescales with different time resolutions. Most of our experiments were carried out using a time resolution of 15 sec with a total imaging duration of 225 sec (~4 min). At this timescale, the MPS appeared dynamic along much of the length of axons, where stretches of the MPS disassembled and re-assembled, often over multiple cycles (Fig 1B). We observed similar dynamics for other structural components of the MPS by imaging neurons expressing GFP-adducin or F-actin labeled with SiR-actin (40) (fig. S1). We confirmed that the observed cycles of disappearance and reappearance of the MPS signal were not due to local movements of the axon or bulk flow of the membrane by co-imaging with the membrane marker, CellBrite, which showed that axons stayed in focus as the MPS disassembled (Fig. 1C and fig. S2).

We also performed SIM imaging of spectrin-mNeonGreen at a faster time resolution of 1 sec. However, photobleaching limited our acquisition to ~20 total timepoints in SIM imaging. At this timescale, we observed disappearance (or substantial reduction in intensity) and reappearance of individual MPS rings (Fig. 1D), although such events were infrequent, with relatively few events observed due to the short 20-sec imaging duration. At the other end of the spectrum, to gain insight into the extent of MPS remodeling over longer timescales, we increased the imaging duration to 1 hour by reducing the time resolution to 5 min. In this regime, we observed more dramatic examples of MPS remodeling, where long stretches of MPS disappeared and reappeared (Fig. 1E; fig. S3), but individual transitions were often not well-resolved due to the low time resolution.

Overall, the 15-sec time resolution provided a good balance between resolving individual MPS transitions and capturing a sufficient number of transitions to quantify the prevalence of remodeling. Our quantifications showed that MPS remodeling occurred in > 80% of all axons observed and covered 20–30% of the length of axons during the ~4 min imaging window (Fig. 1F and fig. S1, C and F). At any given time, only a small fraction of the MPS underwent remodeling, often exhibiting repeated cycles of local disappearance and reappearance, suggesting that regions of the MPS transition from a relatively long-lived static state to a short-lived labile state in which it undergoes cycles of degradation and reformation. MPS remodeling primarily occurred in the medial and distal regions of axons and rarely in regions proximal to the cell body (fig. S4A). When we co-labeled synaptic sites during live-cell imaging of the MPS, we found that most of the observed remodeling events occurred outside of synapses, but the percentage of axonal length exhibiting remodeling appeared to be comparable both within and outside of synaptic sites (fig. S4, B to D).

The fact that we observed similar remodeling not only with fluorescent fusion proteins of βII-spectrin and adducin, but also with the small-molecule F-actin-binding dye, SiR-actin, suggests that such dynamics are unlikely to be caused by the fluorescent reporters. Moreover, our observations of constitutive MPS remodeling suggest that, in a snapshot, a small fraction of the MPS should exhibit imperfections arising from the dynamics, which should be visible in fixed-cell immunofluorescence images that are free from potential perturbation by fluorescent reporters in live cells. Indeed, super-resolution STORM images of fixed neurons showed that ~30% of axonal length was covered by imperfect MPS (fig. S5), consistent with our live-cell imaging results. Because mNeonGreen labeling of endogenous spectrin allows uniform labeling and clear visualization of the MPS without being masked by other actin structures or potential artifacts caused by overexpression of MPS components, offering a convenient and robust way to observe MPS remodeling, we used this labeling scheme for quantitative and mechanistic characterizations of MPS dynamics below.

## Calcium signaling drives MPS remodeling

Next, we investigated the mechanisms underlying MPS remodeling. Because many different processes could contribute to MPS remodeling, we first used conventional diffraction-limited imaging, which has a higher throughput than super-resolution imaging, to perform an initial screen. Although mNeonGreen fluorescence was largely uniform in these conventional images because the periodic spacing of the MPS (~180–190 nm) is below the diffraction limit, we reasoned that when stretches of the MPS disassemble and reassemble, we should observe the disappearance and reappearance of the spectrin signal in segments of the axons. Using this approach, we could more rapidly screen perturbations that potentially affect MPS dynamics, which could then be validated by super-resolution imaging.

**Fig. 2:**
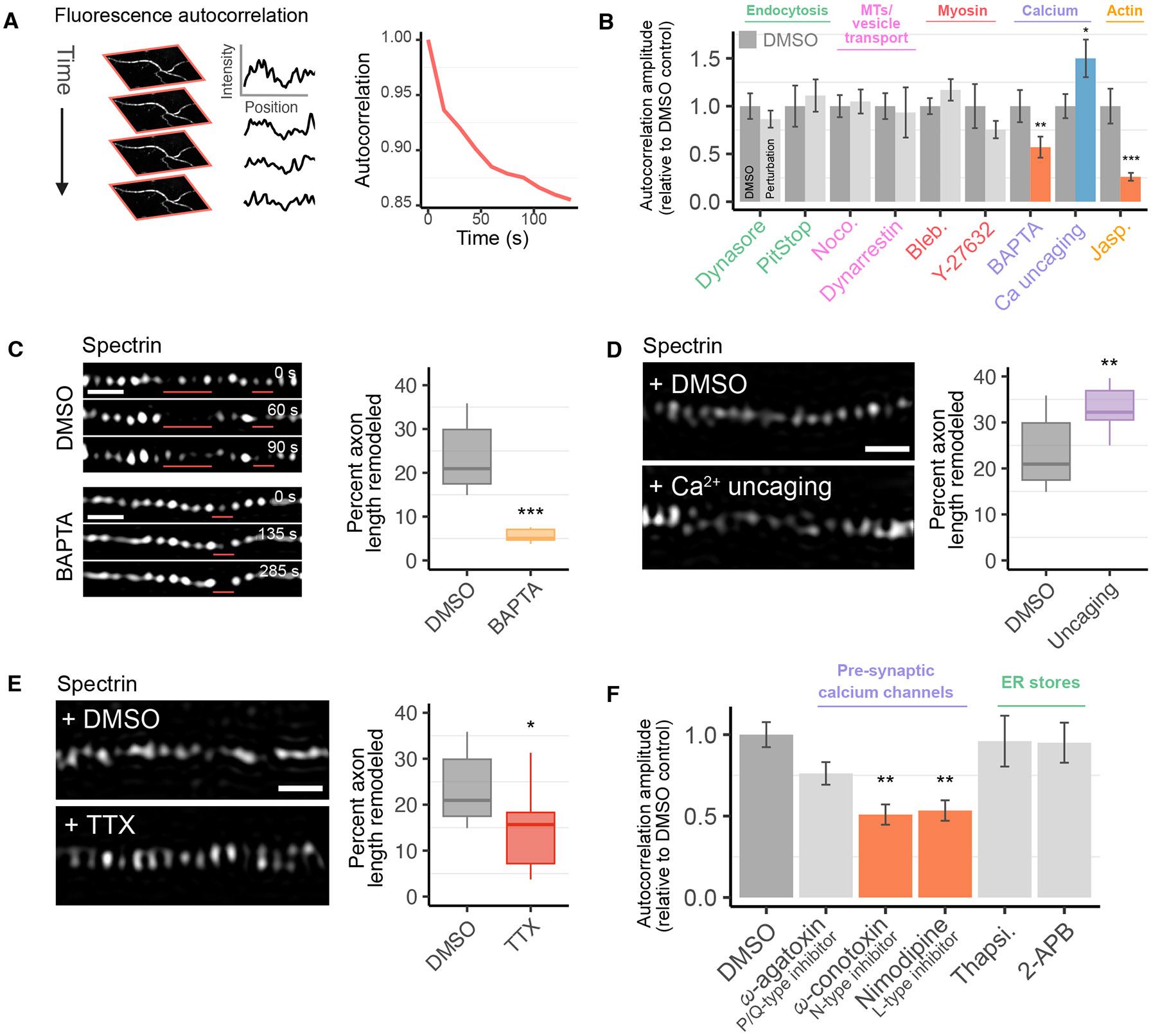
Calcium signaling drives MPS remodeling. (**A**) Schematic of fluorescence autocorrelation analysis used to quantify spectrin dynamics in diffraction-limited imaging. *Left*, spectrin-GFP intensity changes along the axon in consecutive frames of imaging. *Right*, for each axon, intensity changes as a function of time are quantified by an autocorrelation function, whose amplitude reflects the prevalence of remodeling. (**B**) Screen of potential regulators of MPS remodeling using diffraction-limited imaging. Average autocorrelation amplitudes normalized to the DMSO control are plotted. (**C**) *Left*, snapshots from representative live SIM imaging of spectrin-mNeonGreen in axons treated with 10 *μ*M of the Ca^2+^ chelator, BAPTA, or DMSO control 30 min prior to imaging. Remodeling regions are indicated with red lines. *Right*, fraction of axon length exhibiting MPS remodeling over the 225-sec imaging window. Experiments in panels C–E were performed together with a shared DMSO control. (**D**) *Left*, representative snapshots of spectrin-mNeonGreen in axons treated with 10 *μ*M caged Ca^2+^ (NP-EGTA, lower panel) or DMSO. Continuous low-intensity 405 nm illumination was used to induce Ca^2+^ uncaging at a relatively low degree. *Right*, fraction of axonal length exhibiting MPS remodeling. (**E**) *Left*, representative snapshots of spectrin-mNeonGreen in axons treated with 1 *μ*M TTX, to silence neuronal activity, or DMSO control 30 min prior to imaging. *Right*, fraction of axonal length exhibiting MPS remodeling. (**F**) Measurement of MPS dynamics in diffraction-limited imaging of spectrin-mNeonGreen expressing neurons treated with inhibitors of P/Q-type Ca^2+^ channels (ω-agatoxin IVA), N-type Ca^2+^ channels (ω-conotoxin), and L-type Ca^2+^ channels (nimodipine), as well as inhibitors of Ca^2+^ uptake (thapsigargin or Thapsi) or Ca^2+^ release (2-APB) from ER stores. Average autocorrelation amplitudes normalized to DMSO control are plotted. Statistics for (B-F) are shown in Table S2. Scale bars in C-E, 500 nm.

As expected, diffraction-limited imaging showed disappearance (or substantial reduction in intensity) and reappearance of spectrin-mNeonGreen signal in stretches of axons in cultured hippocampal neurons (fig. S6A). To quantify these dynamics, we computed the autocorrelation function of the fluorescent signal over time in time-lapse diffraction-limited images of axons (Fig. 2A and fig. S6B). The amplitude of this autocorrelation function measures the extent to which the fluorescence signal changes and hence reflects the prevalence of MPS remodeling: the more prevalent the remodeling, the higher the autocorrelation amplitude. As controls, we transduced neurons with a cytoplasmic GFP or labeled the neuronal membrane with CellBrite. In both cases, the autocorrelation amplitude was substantially smaller than that observed for spectrin-mNeonGreen (fig. S6B), where the small residual fluctuations could be due to small movements of the axon or the flow of proteins and lipids (42).

Using this approach, we screened several intracellular processes that are potentially linked to spectrin, actin, and membrane remodeling using pharmacological inhibitors and activators. These processes include (i) clathrin-dependent and -independent endocytosis, which may remodel or perturb membrane structures; (ii) microtubules, which could interact with the MPS (39), and microtubule-dependent transport, and in particular, dynein–dynactin-driven microtubule transport, in which spectrin plays a pivotal role (43); (iii) myosin-II, which has been shown to be associated with actin filaments in the MPS (15, 28, 29); (iv) calcium signaling, which can regulate cytoskeletal modeling and spectrin degradation (44); and (v) actin polymerization and depolymerization, which can affect the integrity of the MPS (9, 10).

Our screen showed that inhibitors of endocytosis (PitStop and dynasore), dynein-dependent transport on microtubules (dynarrestin), the actin-dependent motor myosin-II (blebbistatin or Bleb) and its upstream regulator, ROCK (Y-27632), as well as a microtubule depolymerizing drug (nocodazole or Noco), did not have substantial effects on spectrin dynamics, as reflected by the autocorrelation amplitude of the spectrin signal (Fig. 2B). In stark contrast, perturbation of intracellular Ca^2+^ concentration and actin stability had a strong effect on spectrin dynamics. Reducing intracellular Ca^2+^ concentration with the Ca^2+^ chelator, BAPTA, substantially reduced spectrin dynamics (Fig. 2B). Concordantly, increasing intracellular Ca^2+^ concentration using the photo-uncaging of a cell-permeable caged Ca^2+^ compound, NP-EGTA (45), markedly increased spectrin dynamics (Fig. 2B). In addition, spectrin dynamics were substantially reduced by jasplakinolide (Jasp), a drug that stabilizes actin filaments, suggesting that actin polymerization/depolymerization also play a role in MPS dynamics.

Although diffraction-limited imaging is efficient in screening a large number of potential candidate factors, direct observation of the structure and dynamics of the MPS requires super-resolution imaging. Having uncovered intracellular Ca^2+^ as a potential regulator of MPS dynamics, we therefore returned to live-cell SIM imaging of the MPS, using spectrin-mNeonGreen as a marker, to directly probe and quantify the effects of these perturbations. Compared to control samples treated with DMSO, treatment of neurons with the Ca^2+^ chelator, BAPTA, drastically reduced the prevalence of MPS remodeling, quantified as the fraction of axon length exhibiting MPS disassembly and reassembly over the course of our 225-sec imaging window (Fig. 2C). Concordantly, continuous low-level uncaging of Ca^2+^ increased the prevalence of MPS remodeling (Fig. 2D). Both results are in agreement with our screen using diffraction-limited imaging.

## Neuronal activity promotes MPS dynamics

Because action potentials generated by neuronal activity are a primary driving force for Ca^2+^ influx in neurons, we next used tetrodotoxin (TTX) to block action potentials and tested the effect of this drug on MPS remodeling. With TTX treatment, we observed a reduction in the prevalence of MPS remodeling (Fig. 2E), but not at the magnitude of direct chelation of intracellular Ca^2+^ by BAPTA (Fig. 2C), suggesting that neuronal activity can promote, but is not the sole factor driving, MPS remodeling.

The means by which TTX influences MPS remodeling is likely through the inhibition of intracellular Ca^2+^ spiking activity associated with action potentials. However, because the magnitude of the TTX effect was substantially lower than that of Ca^2+^ chelation by BAPTA, other sources of Ca^2+^ activity must also play a role in MPS remodeling. Indeed, when we imaged Ca^2+^ spiking in neurons using the Ca^2+^ indicator dye, Cal-520, both TTX and BAPTA abolished transient Ca^2+^ spikes (fig. S7A). However, Ca^2+^ chelation with BAPTA also led to a sustained reduction in resting Ca^2+^ concentration in the neurons, whereas TTX did not affect resting Ca^2+^ concentration (fig. S7B).

To further explore the sources of the Ca^2+^ influx that contribute to MPS remodeling, we measured spectrin dynamics in the presence of various toxins that inhibit voltage-gated Ca^2+^ channels or affect the uptake and release of Ca^2+^ from local ER stores. We found that inhibition of N-type Ca^2+^ channels (by ω-conotoxin) and L-type Ca^2+^ channels (by nimodipine) both led to a reduction in spectrin dynamics (Fig. 2F; fig. S7). Perturbing ER calcium stores (inhibiting Ca^2+^ uptake into the ER with thapsigargin or Ca^2+^ release from the ER with 2-APB), on the other hand, had little effect on MPS dynamics (Fig. 2F). These results suggest that Ca^2+^ influx through voltage-gated Ca^2+^ channels, encompassing both action potential-induced depolarization and sub-threshold depolarization from spontaneous synaptic activity, are likely sources of Ca^2+^ driving MPS dynamics. However, other sources of local Ca^2+^ likely also contribute to MPS dynamics. We also observed that Ca^2+^ channels exhibited a colocalization with the MPS (fig. S8), which could potentially facilitate the rapid effect of Ca^2+^ influx on MPS remodeling.

## MPS dynamics is promoted by multiple Ca^2+^-dependent enzymatic processes

**Fig. 3:**
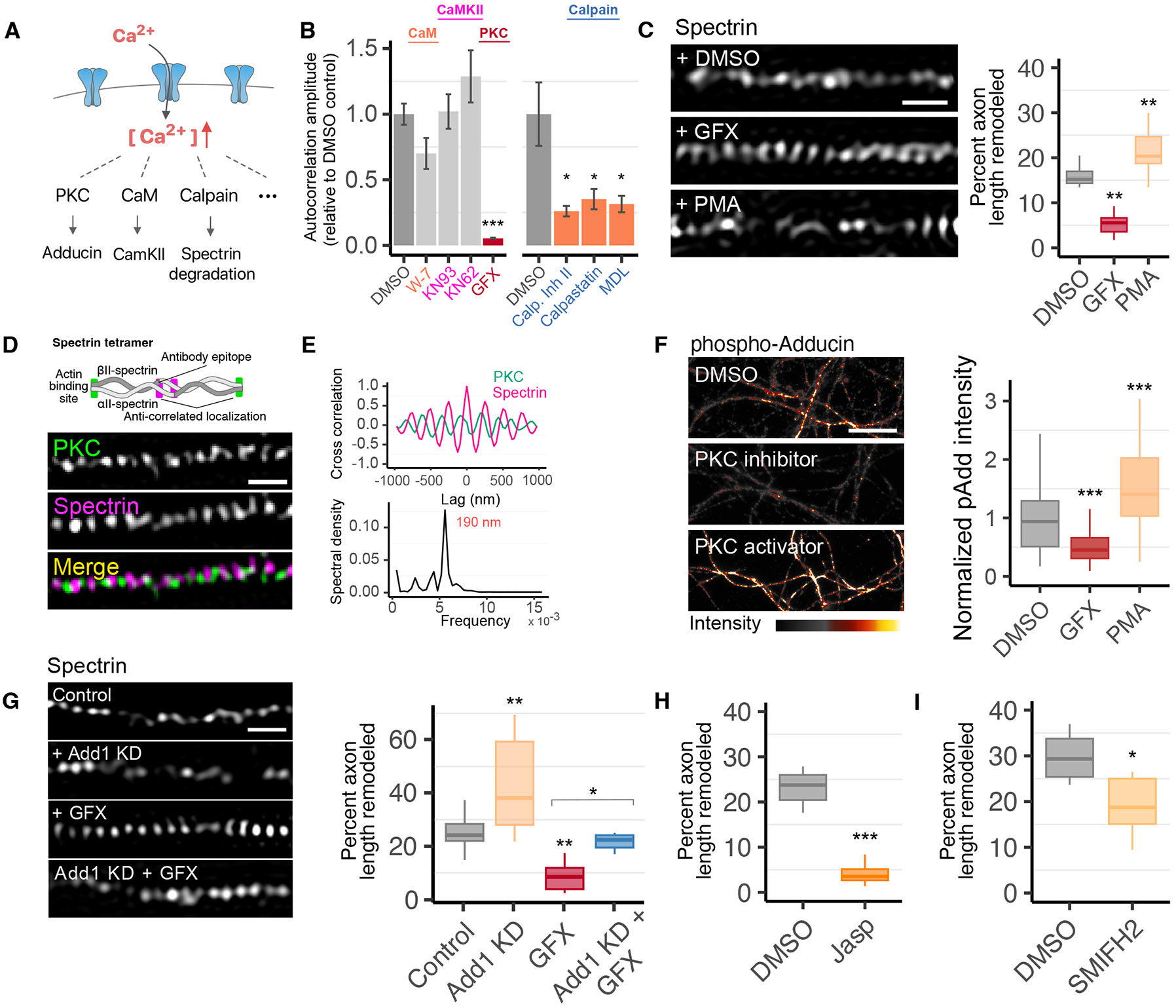
PKC-mediated adducin phosphorylation promotes MPS remodeling. (**A**) Schematic of several Ca^2+^ signaling pathways that may act on the MPS. (**B**) Screen of several major effectors of calcium signaling in MPS remodeling by diffraction-limited imaging. *Left*, W-7: calmodulin inhibitor; KN93 and KN62: CamKII inhibitors; GFX: PKC inhibitor. *Right*, multiple calpain inhibitors. (**C**) *Left*, representative snapshots of spectrin-mNeonGreen in axons treated with 10 *μ*M of the PKC inhibitor, GFX, 1 *μ*M of the PKC activator, PMA, or DMSO control 30 min prior to SIM imaging. *Right*, fraction of axon length exhibiting MPS remodeling. (**D**) Two-color SIM imaging of PKC and spectrin in fixed neurons, illustrating the periodic distribution of PKC in an anticorrelated manner with that of spectrin in some regions of axons. Schematic shows the spectrin epitope and actin binding sites. (**E**) *Top*, cross-correlation analysis of PKC (green) and spectrin (magenta) in two-color SIM images. The average cross-correlation of 7 axonal regions exhibiting high PKC periodicity is plotted. *Bottom*, spectral analysis of the average autocorrelation of 27 axonal regions from 3 FOVs showing a periodic distribution of PKC with a 190-nm interval. (**F**) Measurement of adducin phosphorylation by PKC in axons. Representative images of fluorescence intensity (left) and quantification of normalized intensity (right) of phosphorylated adducin signal in axons following PKC inhibition with 10 *μ*M GFX or activation using 1 *μ*M PMA as compared to DMSO control. (**G**) *Left*, representative snapshots from live SIM imaging of spectrin-mNeonGreen neurons treated with control shRNA, shRNA targeting adducin 1 (Add 1 KD), GFX to inhibit PKC, or adducin shRNA together with GFX. *Right*, fraction of axon length exhibiting MPS remodeling under these conditions. (**H**) Fraction of axon length exhibiting MPS remodeling in neurons treated with 10 *μ*M Jasp to stabilize actin filaments or DMSO control for 30 min prior to live SIM imaging. These experiments were performed together with those in Fig. 4A and 4G with a shared DMSO control. (**I**) Fraction of axon length exhibiting MPS remodeling in neurons treated with 30 *μ*M of the formin inhibitor, SMIFH2, or DMSO control for 10 minutes prior to imaging. Statistics for (B, C, F-I) are shown in Table S2. Scale bars, 500 nm (C, D, and G); 10 *μ*m (F).

The results described above indicate that Ca^2+^ plays a primary role in MPS dynamics. We next investigated the potential molecular mechanisms underlying Ca^2+^-driven MPS remodeling. To this end, we first used diffraction-limited imaging to screen the effects of several calcium-dependent enzymes that can potentially act on MPS components (Fig. 3A). These include (i) calpain, a Ca^2+^-dependent protease that can cleave spectrin and actin (46); (ii) protein kinase C (PKC), a Ca^2+^-dependent kinase that can phosphorylate adducin (47, 48); and (iii) calmodulin (CaM), the Ca^2+^-bound form of which interacts with Ca^2+^/CaM-dependent protein kinase II (CaMKII) (49), which is associated with the MPS (15). Spectrin and adducin also contain CaM-binding domains that regulate the stability of actin-spectrin complexes (50-52). We found that inhibition of PKC using GF 109203X (GFX) and inhibition of calpain using MDL 28170 (MDL), calpastatin, or calpain inhibitor II strongly reduced spectrin dynamics, measured by autocorrelation analysis of time-lapsed diffraction-limited images, whereas inhibition of CaM with the inhibitor, W-7, or CamKII with the inhibitors KN93 and KN62 had no appreciable effect (Fig. 3B). Other Ca^2+^-dependent processes may also contribute to MPS dynamics, among them the Ca^2+^-dependent conformational change of the EF-hand motifs in spectrin, which alters the strength of its interaction with actin (53-55), although we did not test the EF-hand effect in this work.

## Ca^2+^-dependent PKC activity promotes MPS dynamics

PKC is a major Ca^2+^-dependent effector of signaling at the membrane (47, 56) that is also known to phosphorylate adducin, a component of the MPS (9, 48, 57). To further validate that PKC inhibition affects MPS remodeling, we imaged spectrin-mNeonGreen knock-in neurons using live-cell SIM imaging to directly resolve the MPS structure and observed that PKC inhibition by GFX drastically reduced the prevalence of MPS remodeling (Fig. 3C). Concordantly, enhancement of PKC activity by phorbol 12-myristate 13-acetate (PMA, a PKC activator) caused a substantial increase in MPS dynamics (Fig. 3C and fig. S9). Together, these results highlight PKC as a major effector of Ca^2+^ in mediating MPS remodeling.

Next, we explored the phosphorylation targets of PKC that could affect MPS dynamics. Although phosphorylation of spectrin could regulate its stability in the membrane (58), PKC is not known to directly phosphorylate spectrin. Among the most likely targets of PKC in the MPS is adducin, which caps actin filaments in the actin rings of the MPS and interacts with both actin and spectrin, serving as a linker that strengthens actin-spectrin interactions in many cell types (57, 59). Adducin phosphorylation by PKC is known to result in adducin dissociation from actin and to diminish its ability to recruit spectrin, reducing actin-spectrin complex stability (48, 57). We thus tested whether PKC-induced phosphorylation of adducin is involved in MPS remodeling.

First, we directly imaged PKC together with spectrin to examine whether PKC is associated with the MPS. We imaged βII-spectrin using an antibody that targets its C-terminus, which is located at the midpoint of the spectrin heterotetramer connecting actin rings of the MPS (Fig. 3D, schematic). Two-color SIM imaging revealed that PKC adopts a periodic distribution in some axonal regions, localizing to sites midway between bands of spectrin, with the two being anti-correlated with each other (Fig. 3, D and E). These results suggest that PKC is associated (likely transiently) with the actin rings and point to adducin as the potential target of PKC in the MPS. Further supporting this notion, we found that inhibition of PKC by GFX decreased phospho-adducin signal in axons, whereas activation of PKC by PMA had the opposite effect, increasing phospho-adducin signal (Fig. 3F), indicating that PKC indeed regulates adducin phosphorylation in neurons.

To test whether adducin plays a role in MPS remodeling, we depleted adducin from neurons using a virally delivered shRNA against adducin. Because the MPS is degraded in neurons from adducin knock-out animals (15), we transduced neurons with shRNA at concentrations that maintain a reasonable degree of MPS integrity (fig. S10). Under these conditions, we observed a substantial increase in the prevalence of MPS remodeling (Fig. 3G). Finally, we tested whether adducin knockdown could counter the reduction in MPS dynamics caused by PKC inhibition. We observed that, although PKC inhibition by GFX drastically reduced MPS dynamics, adducin knockdown largely compensated for this effect when we combined GFX treatment with adducin knockdown (Fig. 3G). Taken together, these results suggest that phosphorylation of adducin by PKC, which could reduce actin filament stability and destabilize actin-spectrin interactions, promotes MPS remodeling, although we cannot rule out the possibility that phosphorylation of other targets by PKC may also contribute to MPS remodeling.

## Actin-filament stability and dynamics play a role in MPS remodeling.

The striking effect of perturbing PKC and adducin suggests that stability of the actin rings plays an important role in MPS dynamics. Indeed, our diffraction-limited imaging and SIM imaging of the MPS both indicated that stabilization of actin filaments with Jasp resulted in a drastic reduction in the prevalence of MPS remodeling (Figs. 2B and 3H). This stabilization by Jasp could occur after PKC-mediated adducin phosphorylation, which uncaps actin filaments and makes them prone to depolymerization. It is also possible that a certain degree of basal actin depolymerization occurs independently of PKC or Ca^2+^ signaling, which is inhibited by Jasp.

In addition to PKC-induced adducin phosphorylation, we wondered whether other factors that regulate actin filaments could also influence MPS dynamics. We therefore screened two major families of actin filament nucleation/elongation factors for a potential effect on MPS dynamics using diffraction-limited imaging. This included mediators of branched actin (60) and linear actin (61, 62) formation. We observed that inhibition (by CK666) of Arp2/3, a nucleator of branched actin filaments (63), did not affect MPS dynamics appreciably (fig. S11A), consistent with previous observations that MPS is not substantially disrupted by Arp2/3 inhibition (39). On the other hand, short-term inhibition (10 minutes, by SMIFH2) of formin, a nucleator and polymerizer of linear actin filaments (64), substantially reduced MPS dynamics (fig. S11A). To validate this result, we performed live-cell SIM imaging of the MPS in neurons treated with SMIFH2 and found that short-term formin inhibition indeed resulted in a moderate reduction in the prevalence of MPS remodeling (Fig. 3I).

The formin family of linear actin nucleators have previously been implicated in MPS maintenance (39). Consistent with this work, we found that the formins mDia2, Daam1, and Fmn2 were associated with the actin rings of the MPS (fig. S11, B to G), albeit only in isolated stretches of the axon, suggesting that their association with the MPS is transient or local. Long-term (3 hour) inhibition of formins with the inhibitor SMIFH2 as well as shRNA-mediated knockdown of mDia2 both resulted in partial, moderate loss of the MPS (figs. S12 and S13). Our results thus suggest that formins have a dual role in MPS maintenance and dynamics. Mechanistically, since both formin and adducin bind to the barbed end of actin filaments, formin binding could displace adducin from actin filaments (65-67), leading to MPS destabilization and promoting remodeling, because adducin binds to both actin and spectrin and stabilizes the actin-spectrin complex (48, 57, 59). Thus, short-term inhibition of formin could lead to increased adducin binding to actin filaments, stabilizing the actin-spectrin complex and the MPS. In the long term, however, formin inhibition could slowly deplete the axon of actin filament supplies, resulting in a reduced ability to repair the MPS after its disassembly.

## Ca^2+^-dependent spectrin proteolysis by calpain promotes MPS dynamics

**Fig. 4.**
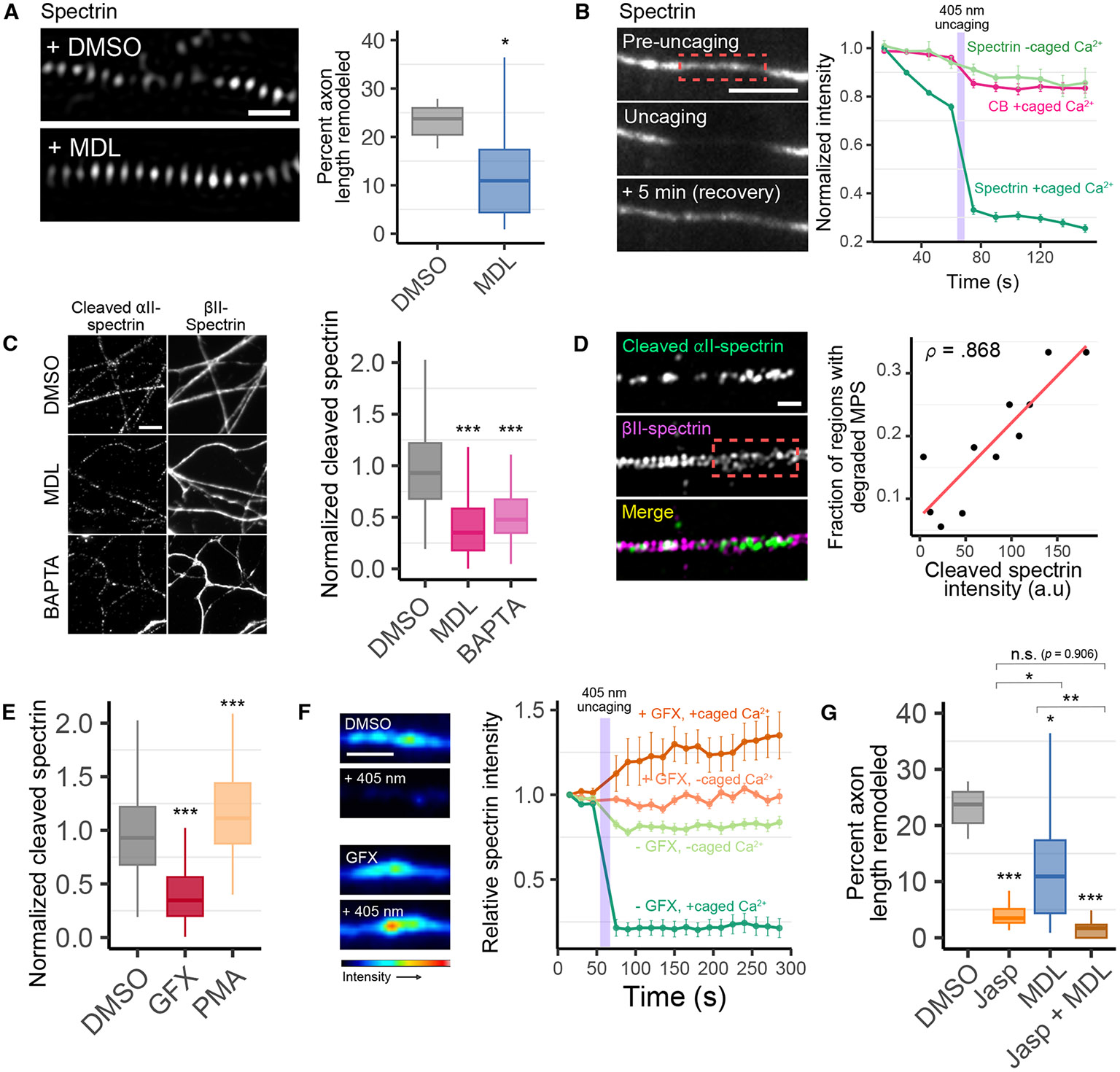
Calpain-dependent spectrin proteolysis promotes MPS remodeling. (**A**) *Left*, snapshots of spectrin-mNeonGreen in axons treated with 50 *μ*M MDL, a calpain inhibitor, or DMSO control 30 min prior to imaging. *Right*, fraction of axonal length exhibiting MPS remodeling (sharing DMSO control with experiments in Fig. 3H and 4G). (**B**) *Left*, snapshots of diffraction-limited imaging of spectrin-mNeonGreen during an intense pulse of 405-nm illumination to induce Ca^2+^ uncaging in a small region of an axon treated with 30 *μ*M NP-EGTA. Upper: prior to the 405-nm illumination. Middle: immediate after the 405-nm pulse. Lower: 5 minutes after the 405-nm pulse. *Right*, dark green: normalized spectrin-mNeonGreen intensity before and after Ca^2+^ uncaging with the time of the 405-nm uncaging pulse indicated by the purple bar. The moderate signal decrease before the 405-nm pulse is due to Ca^2+^ uncaging by the 488-nm light used to image spectrin-mNeonGreen. Light green: same as dark green but without caged Ca^2+^ (NP-EGTA). Magenta: Same as dark green but for the intensity of CellBright (CB), a plasma membrane marker. (**C**) Co-immunofluorescence imaging of βII-spectrin and calpain-cleaved αII-spectrin in neurons treated with MDL, BAPTA, or DMSO control. Quantification of calpain-cleaved αII-spectrin is shown on the right. (**D**). *Left*, Two-color SIM imaging of βII-spectrin and calpain-cleaved αII-spectrin. Dash-boxed region shows regions of high calpain activity corresponding to areas of degraded MPS. *Right*, correlation between fraction of axonal regions exhibiting degraded MPS and calpain-cleaved αII-spectrin intensity. (**E**) Cleaved αII-spectrin intensity in axons following PKC inhibition (10 *μ*m GFX) or activation (1 *μ*M PMA) as compared to control. (**F**) Effect of GFX on spectrin degradation in response to Ca^2+^ uncaging. *Left*, images of spectrin-mNeonGreen fluorescence in axons before and after an intense pulse of 405-nm light to induce Ca^2+^ uncaging in neurons pre-treated with 30 *μ*M NP-EGTA and either 10 *μ*M GFX (bottom panels) or DMSO control (top panels) 30 minutes prior to imaging. *Right*, spectrin intensity before and after the 405-nm uncaging pulse (purple bar) in the absence and presence of GFX. Control without caged Ca^2+^ are also shown. (**G**) Fraction of axonal length exhibiting MPS remodeling in neuron treated with Jasp, MDL, or both. Statistics for (A-G) are shown in Table S2. Scale bars, 500 nm (A and D), 5 *μ*m (B and C), 2 *μ*m (F).

Our screen results (Fig. 3B) suggest that, in addition to PKC, Ca^2+^-dependent proteolysis by calpain also contributes to spectrin dynamics. To validate this, we performed live-cell SIM imaging of the MPS in spectrin-mNeonGreen knock-in neurons and found that inhibition of calpain by MDL indeed substantially reduced the prevalence of MPS remodeling (Fig. 4A).

Spectrin is a target of the Ca^2+^-dependent protease calpain, and calpain-mediated proteolysis has the potential to regulate membrane skeleton stability (44, 68). We thus asked whether spectrin proteolysis in response to Ca^2+^ contributes to MPS remodeling. To this end, we first examined the effects of applying brief pulses of Ca^2+^ to local regions of the axon using UV light to uncage a relatively high concentration of the caged Ca^2+^ compound, NP-EGTA. Immediately following uncaging, we observed a rapid, drastic reduction in spectrin fluorescence (Fig. 4B), corresponding to MPS degradation (fig. S14). Upon removal of the uncaging laser, the spectrin signal recovered within 5 minutes (Fig. 4B). This suggests that local increases in Ca^2+^ could degrade spectrin and the MPS.

We next probed the involvement of calpain-mediated spectrin degradation in MPS remodeling by directly imaging calpain-cleaved spectrin in axons. To this end, we performed SIM imaging of fixed neurons co-immunostained with an antibody targeting βII-spectrin and an antibody that specifically targets the calpain-cleaved form of αII-spectrin under unstimulated conditions. Cleaved spectrin was observable throughout the axon (Fig. 4C). The amount of cleaved spectrin was substantially reduced following either calpain inhibition by MDL or Ca^2+^ chelation with BAPTA (Fig. 4C), confirming that the measured signal is indeed due to calpain-dependent cleavage and that this process is Ca^2+^ dependent. We observed a strong correlation between the cleaved αII spectrin signal and the prevalence of MPS degradation (Fig. 4D). Together, our results suggest that calpain-mediated spectrin proteolysis plays a role in MPS remodeling downstream of Ca^2+^.

## PKC activity enhances spectrin degradation by calpain

Since PKC-induced phosphorylation of adducin and calpain-mediated cleavage of spectrin both contribute to MPS remodeling, we wondered whether these two activities are connected. To explore this, we first tested the effects of the PKC inhibitor, GFX, and the PKC activator, PMA, on spectrin cleavage by calpain in unstimulated cells. We found that calpain-cleaved αII-spectrin was reduced by PKC inhibition and increased by PKC activation (Fig. 4E), suggesting that PKC activity enhances spectrin cleavage by calpain. We next asked whether PKC inhibition could prevent the rapid degradation of spectrin following the influx of Ca^2+^. We found that, after PKC inhibition, spectrin became resistant to degradation even in the presence of a strong Ca^2+^ uncaging pulse (Fig. 4F). Together, these results suggest the possibility that adducin phosphorylation by PKC, which promotes the dissociation of adducin and weakens the link between actin and spectrin, can enhance the accessibility of the spectrin cleavage site and thereby facilitate spectrin degradation by calpain. However, our results do not exclude the possibility that some spectrin cleavage by calpain could also occur in a PKC-independent manner.

The results above, as well as the fact that the effect of PKC inhibition on MPS dynamics is stronger than that of calpain inhibition by MDL, suggest that spectrin degradation might play a secondary role in MPS dynamics as compared to actin-ring destabilization. To further test this possibility, we compared the effects of stabilizing actin by an alternative means, with Jasp, to that of calpain inhibition with MDL. We found that the effect of Jasp was substantially stronger than that of MDL (Fig. 4G). In addition, the combined effect of both drugs was substantially stronger than that of MDL alone but not statistically significantly (p-value = 0.9) stronger than that of Jasp alone (Fig. 4G), supporting the notion that calpain-medicated spectrin cleavage is at least in part dependent on actin destabilization.

Taken together, our results suggest that actin-ring destabilization caused by PKC activity is the primary driver of MPS dynamics, whereas spectrin degradation occurs downstream and also promotes MPS remodeling. However, our results do not exclude the possibility that some spectrin degradation may also occur independently of actin-ring destabilization.

## MPS remodeling promotes endocytosis

Our discovery of a highly regulated system of MPS remodeling implies that MPS dynamics are functionally important. Recent studies have shown that the MPS inhibits clathrin-mediated endocytosis of GPCRs upon ligand simulation (23) and restricts endocytosis in the AIS under resting conditions (69). Given the unique form that MPS dynamics takes, in which an apparently persistent scaffold is repeatedly disassembled and rebuilt locally, we hypothesized that the presence of the MPS could inhibit cellular processes that require local membrane access or deformation, such as endocytosis, and that the MPS must be temporarily and locally removed to facilitate these processes while remaining largely intact to support other MPS functions.

**Fig. 5.**
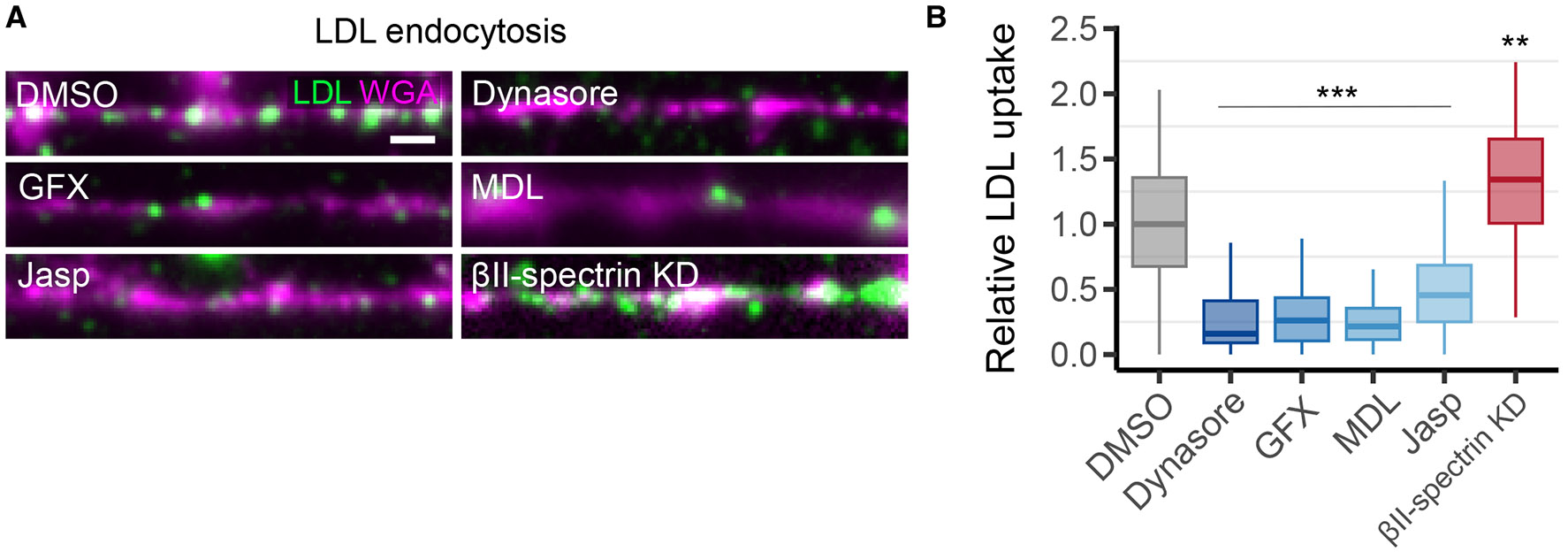
MPS dynamics promote endocytosis. (**A**) Representative images of low-density lipoprotein (LDL, 5 *μ*g/mL) uptake in axons following a 90 min incubation in unstimulated neurons (LDL in green with the axonal membrane counterstained with WGA in magenta). *Top*, Treatment of neurons with the clathrin-mediated endocytosis inhibitor (dynasore, right) and DMSO control (left) for 30 min prior to LDL uptake. *Middle and bottom*, LDL uptake following perturbations that stabilize the MPS and reduce MPS dynamics (PKC inhibition with 10 *μ*M GFX, calpain inhibition with 50 *μ*M MDL, and actin stabilization with 10 *μ*M Jasp) or a perturbation that removes the MPS (*β*II-spectrin knockdown). (**B**) Quantification of LDL uptake in axons under the conditions shown in (A). Statistics for (B) are shown in Table S2. Scale bar in (A), 1 *μ*m.

To explore whether MPS remodeling serves to facilitate endocytosis, we measured basal endocytosis in axons and tested the effects of various perturbations that impact MPS dynamics. First, we measured the uptake of low-density lipoprotein (LDL) as a reporter of receptor-mediated endocytosis in unstimulated neurons. We confirmed that the measured fluorescence signal of LDL in axons was primarily due to clathrin-mediated endocytosis by using dynasore to inhibit endocytosis (Fig. 5, A and B). We then tested the effects of three perturbations shown to reduce MPS dynamics, including PKC inhibition by GFX, calpain inhibition by MDL, and actin stabilization with Jasp, as well as the effect of MPS removal by βII-spectrin knockdown. We found that all three perturbations that stabilize the MPS and reduce MPS remodeling resulted in a substantial decrease in endocytosis, whereas removal of the MPS increased endocytosis (Fig. 5, A and B). We further extended these results using the lipophilic dye, AM5-65, as a general reporter of endocytosis (fig. S15), indicating that these effects are not limited to the endocytosis of a particular ligand or receptor.

Although some of the individual perturbations that we employed are not specific to the MPS and could affect endocytosis by altering other cellular structures or processes, our observations that all three drugs that stabilize the MPS (GFX, MDL, and Jasp) similarly inhibited endocytosis support the notion that MPS dynamics facilitate endocytosis. Furthermore, unlike the other perturbations, βII-spectrin is predominantly localized to the MPS in axons, and the observation that endocytosis in axons was increased following knockdown of βII-spectrin also supports the notion that disassembly of the MPS enhances endocytosis. The increase in endocytosis was only ~30% when the MPS is removed, whereas the reduction in endocytosis upon inhibition of MPS dynamics was much more substantial (70–80%), suggesting that the MPS imposes a strong inhibitory effect on endocytosis and that the constitutive dynamics of the MPS is sufficient to largely remove this inhibition.

## Discussion

In summary, using live-cell super-resolution imaging, we made the unexpected discovery that the MPS is constitutively dynamic, exhibiting repeated cycles of local disassembly and reassembly even under unstimulated conditions. These dynamics can contribute to remodeling of large portions of the axonal membrane skeleton over the course of minutes to hours. This is particularly surprising in light of the role that the MPS is presumed to play in providing axons with mechanical support and the resistance of the MPS to degradation by pharmacological perturbations.

Our studies provide mechanistic insight into a highly regulated system of MPS remodeling (Fig. S16). The primary driver of MPS remodeling is Ca^2+^ signaling, which activates multiple pathways that intersect in the process. Ca^2+^-dependent activation of PKC leads to adducin phosphorylation, destabilizing the actin ring and weakening actin-spectrin interactions. At the same time, Ca^2+^ also activates calpain, leading to proteolysis of spectrin. Together, these activities cause the local disassembly of the MPS. The two pathways intersect in their contributions to MPS remodeling: PKC activity enhances the cleavage of spectrin by calpain and is required for the degradation of spectrin in response to a large influx of ca^2+^ in uncaging experiments. Mechanistically, we reason that PKC-mediated phosphorylation of adducin, which promotes adducin dissociation, weakens actin-spectrin interactions and increases the accessibility of spectrin to cleavage by calpain.

Although our results have uncovered many major players regulating MPS dynamics, other Ca^2+^-dependent mechanisms may also contribute. For example, adducin has been shown to be susceptible to calpain-dependent proteolysis in a phosphorylation dependent manner in platelets (70). It is thus possible that adducin proteolysis may also contribute to MPS remodeling in axons. In addition, the EF-hand motifs of spectrin are known to undergo a Ca^2+^-dependent conformational change that alters the strength of its interaction with actin (53-55), which may also contribute to MPS remodeling. Moreover, we have identified regulators of actin stability and filament polymerization, including members of the formin family, as contributors to MPS dynamics. It has been shown that, in addition to adducin, genetic perturbation or inhibition of other actin-binding proteins, such as tropomyosin, tropomodulin, and dematin, can also cause MPS disruption to varying degrees (15, 71). It is thus possible that other actin regulators may also work together with the regulators we identified here to maintain a constitutive state of MPS turnover.

Functionally, we found that MPS remodeling facilitates endocytosis in the axon. A defining aspect of the dynamics we have uncovered here is that it involves the repeated, local destruction and rebuilding of an elaborate cytoskeletal structure at the membrane. This is perhaps because the functions of the MPS, such as its role in providing structural stability to long and delicate axons, are at odds with other important functions of the membrane, thus requiring local disruption of the MPS to facilitate these membrane functions. Indeed, we found that the presence of the MPS is inhibitory to endocytosis, and that dynamic, local disruption of the MPS promotes this membrane and cargo internalization process. Such a requirement may also hold for other cellular processes that require deformation of or access to the plasma membrane. Moreover, in MPS’s role as an important signaling platform (23), the disassembly and reassembly dynamics of the MPS could regulate the availability of receptors at the cell surface as well as the proximity of co-receptors and downstream signaling molecules, thereby modulating signaling strength.

Finally, the dynamic nature of Ca^2+^ regulation in neurons suggest many ways that MPS remodeling can be fine-tuned under different conditions to adapt to the needs of the cell. Among them, our observation that inhibition of action potentials and presynaptic Ca^2+^ channels can reduce MPS dynamics suggests an interplay between the MPS and neuronal activity. Understanding the full extent of the functional consequences of MPS remodeling and its interplay with neuronal activity will be an exciting area of future study.

## Materials and Methods

### Generation of βII-spectrin mNeonGreen conditional knock-in mice

Generation of *β*II-spectrin mNeonGreen conditional knock-in (cKI) mice was carried out at the Gene Targeting and Transgenic Facility at the HHMI/Janelia Research campus. The targeting construct, consisting of a LoxP-flanked alternative final exon of *β*II-spectrin with a C-terminal mNeonGreen fusion that is expressed upon Cre expression, was produced using recombineering techniques (72) and traditional molecular cloning. An 8,262 bp genomic DNA fragment containing exons 33–35 of *Sptbn1* was retrieved from BAC clone RP24-63C14. A LoxP site was inserted 188 bp upstream of exon 35, and a polyadenylation signal (3×SV40 PA) was inserted after the 3’ UTR to prevent mNeonGreen leakage before Cre excision. An FRT-NeoR-FRT-LoxP cassette was inserted after 3×PA followed by an exon 35-mNeonGreen fusion (Fig. 1A).

To facilitate embryonic stem (ES) cell targeting, CRISPR/Cas9 genome editing was used. gRNA was *in vitro* transcribed using the MEGA Shortscript T7 kit (ThermoFisher, AM1354), and the template was PCR amplified using the following primers:

*Forward,* 5’- CCTTAATACGACTCACTATAGG GTCTGCGGGTGCAGAGCAGTGGGgttttagagctaGAAAtagc -3’; and

*Reverse,* 5’- AAAAGCACCGACTCGGTGCCACTTTTTCAAGTTGATAACGGACTAGCCTTATTTTAACTTGCTATTTCTAGCTCTAAAAC -3’.

The targeting vector, Cas9 protein (TrueCut Cas9 Protein V2, Fisher Scientific A36499) and the gRNA were co-electroporated into 1 million G1 ES cells derived from F1 hybrid blastocysts of a 129S6 (Taconic, strain 129S6/SvEvTac) × C57BL/6J (JAX, strain C57BL/6J, stock 000664) cross. G4180-resistant ES colonies were isolated and screened by nested PCR using primers outside the construct paired with primers inside the construct. The primers used for ES cell screening were as follows:

5’-arm forward primers:

*Sptbn1-Scr-F1*, 5’- CGCTCACTAGAGCAAAGTTG -3’) and *Sptbn1-Scr-F2.* 5’- TAGGGTTCCTAGTAGGATCC -3’;

5’-arm reverse primers:

*Cre-Scr-R1*, 5’- GAGGGACCTAATAACTTCGT -3’; and *Cre-Scr-R2*. 5’- ATGATCGGAATTGGGCTGCA -3’.

3’-arm forward primers:

*mNeonGreen-Scr-F1,* 5’- CCATAGGATCGAGATCCTGT -3’; and *mNeonGreen-Scr-F2*, 5’- GACTACAACAAGGTCAAGCTGT -3’

3’-arm reverse primers:

*Sptbn1-Scr-R1*, 5’- CAGAGCAGCAGTTCTGACTT -3’; and *Sptbn1-Scr-R2.* 5’- GACCCCAGAGATCTAATTCC -3’.

Eight ES clones with both arms positive were identified for generation of chimeric mice. Chimeric mice were generated by aggregating the ES cells with 8-cell embryos of the CD-1 strain (Charles River, strain code 022). Chimeras were bred for 13 generations with R26FLP females (JAX stock 003946) to remove the FRT-Neo-FRT cassette. Accurate targeting was further confirmed by homozygosity tests in progenies.

### Primary culture of mouse and rat hippocampal neurons

All rodent procedures were approved by the Institutional Animal Care and Use Committee (IACUC) of Harvard University. Primary mouse and rat hippocampal neurons were cultured as follows. For rat neurons, female timed-pregnant CD (Sprague Dawley) rats (Strain code 001, Charles River Laboratories, Wilmington, MA), aged 3–5 months, were euthanized and hippocampi were isolated from E18 embryos in Hibernate A medium without Ca^2+^ (TransnetYX, Cordova, TN). Mouse hippocampi were isolated from P0 pups (a cross between the 129S6 and C57BL/6J strains, mNeonGreen cKI). Following isolation, hippocampi were digested in 0.25% Trypsin-EDTA (1×) (Sigma, T4549) at 37°C for 15 minutes, washed in Hanks’ Balanced Salt Solution (HBSS) (ThermoFisher, 14175079), and transferred to culture medium consisting of Neurobasal media (ThermoFisher, 21103049) supplemented with 2% B27 (ThermoFisher, 17504044), 2 mM GlutaMax (ThermoFisher, 35050-061), and 37.5 mM NaCl. Hippocampi were gently triturated, cells were pelleted at 300 g, resuspended in culture medium, and plated on 18 mm poly-D-lysine-coated coverslips (NeuVitro, GG-18-1.5-pdl) at a density of 80–100k cells per coverslip. Cultures were maintained in a humidity-controlled incubator at 37°C with 5% CO_2_, and half of the media volume was replaced every 5–7 days.

### Adeno- and adeno-associated viral vectors and transduction of primary neurons

Adenoviral vectors expressing Adducin1 shRNA (Ad5-mCherry-U6-rm-ADD1-shRNA), scramble shRNA (Ad5-mCherry-U6-Scrmb-shRNA), mDia2 shRNA (Ad5-mCherry-U6-m-DIAP3-shRNA), and Adducin1-GFP (Ad5-CMV-GFP-Adducin) were custom-ordered from Vector Biolabs (Malvern, PA). The spectrin-GFP adenoviral vector (Ad5-Spectrin-GFP) was custom-ordered from O.D. 260 (Boise, ID). Adenovirus was added to hippocampal cultures between DIV 8–10 to achieve a multiplicity of infection (MOI) of ~20, and transduced neurons were used for experiments 2–7 days later. Cultures of spectrin-mNeonGreen knock-in neurons were transduced at DIV2–3 with Cre recombinase (AAV1-Cre, Vector Biolabs, 7010) at an MOI of 10,000 to induce expression of spectrin-mNeonGreen.

### Antibodies

The following primary antibodies were used in this study: *β*II spectrin (mouse, 1:500, BD Biosciences, 612563, RRID: AB_399853), adducin (rabbit, 1:250, Abcam, ab51130, RRID: AB_867519), phospho-alpha adducin (Ser726, rabbit, 1:250, ThermoFisher, PA5-38064, RRID: AB_2554668), cleavage-specific *α*II-spectrin (a.a. 1171-76, rabbit, 1:250, ECM Biosciences, SP5601), VAMP2 (rabbit, 1:500, Cedarlane, 104202(SY), RRID: AB_887810), DIAPH3/mDia2 (rabbit, 1:200, ThermoFisher, PA5-85667, RRID: AB_2792806), PKC (rabbit, 1:200, Abcam, ab32376, RRID: AB_777294), Daam1 (rabbit, 1:50, ProteinTech, 14876-1-AP, RRID: AB_2089444), Fmn2 (rabbit, 1:50, ThermoFisher, PA5-65632, RRID: AB_2664990), and Ca_v_1.2/CACNA1C (rabbit, Alomone, ACC-130). The following fluorescent dye-conjugated secondary antibodies were used at a dilution of 1:500: donkey anti-rabbit Alexa 488, donkey anti-mouse Alexa 488, donkey anti-mouse Alexa 647, and donkey anti-rabbit Rhodamine Red-X (Jackson Immunoresearch).

### Immunostaining and actin labeling for fixed-cell imaging

Samples were fixed for 15 minutes with 4% paraformaldehyde in PBS with 4% sucrose at room temperature, permeabilized with 0.1% Triton-X 100 for 5 minutes, and blocked for 1 h in buffer consisting of 5% Normal Donkey Serum and 1% BSA in PBS. Primary antibodies were incubated overnight at 4°C, and secondaries for 1 h at room temperature. For labeling of F-actin in two-color SIM imaging, cells were incubated with 1 *μ*M SiR-actin (Cytoskeleton, CY-SC001) or ~1.65 *μ*M phalloidin conjugated with Alexa Fluor 647 for 1 h at room temperature following secondary antibody staining of the other target being imaged together with actin. For labeling the membrane in fixed samples, cells were incubated with 1 *μ*g/mL Alexa Fluor-conjungated WGA (ThermoFisher, W11261) or CellBrite Green (Biotium, 30021) at a dilution of 1:1000. For SIM imaging, coverslips were mounted in ProLong Glass antifade (ThermoFisher, P36982) and allowed to cure overnight. For STORM imaging, buffer consisted of 100 mM Tris, 10 mM NaCl, 5% glucose, and 100 mM cysteamine (Sigma), and an oxygen scavenging system consisting of 0.8 mg/mL glucose oxidase (Sigma) and 40 *μ*g/mL catalase (Roche).

### Super-resolution imaging

Lattice SIM imaging was performed on a Zeiss ELYRA7 super-resolution microscope equipped with an alpha Plan-Apochromat 63x/1.46 Oil objective maintained by the Harvard Center for Biology Imaging (HCBI). Images were acquired and super-resolution images were rendered using Zeiss ZEN software with the recommended SIM processing settings. For fixed samples, 15-phase 3D SIM images were acquired. For live samples, 13 or 9-phase 2D SIM images were acquired and deconvolved using the Sparse-SIM software package (73). The parameters used for Sparse-SIM were: an effective NA of 2.2, 300 iterations of sparsity reconstruction with a sparsity value of 0.1, 30 iterations of Landweber deconvolution, and spatial upsampling.

STORM imaging was performed on a custom-built Olympus IX71 microscope equipped with a 100X oil-immersion objective (Olympus, UPlanSApo, N.A. 1.4) and an EM-CCD camera (Andor iXon, DV887-DCS-BV, Andor Technology). Laser lines including a 405 nm (Coherent, OBIS 405 LX 200 mW), 561 nm (Coherent, Sapphire 561-200 CW CDRH) and 640 nm (Coherent, Genesis MX 639-1000 STM) lasers were aligned to the back focal plane of the objective. A translation stage was used to shift lasers towards the edge of the objective so that the emerging light reached the sample at incidence angles slightly smaller than the critical angle of the glass-water interface, illuminating fluorophores within only a few micrometers of the coverslip surface. A ZT405/488/561/640rpc (Chroma) was used as the dichroic mirror and ZET405/488/561/640mv2 (Chroma) as the emission filter. For 3D STORM imaging, a cylindrical lens was inserted into the detection path so that images of single molecules were elongated along their x or y axes according to their position relative to the focal plane (74). Continuous illumination of 640-nm laser (~2 kW/cm^2^) was used to excite fluorescence from Alexa 647 molecules and switch them into the dark state. Continuous 405 nm excitation (0–1 W/cm^2^) was used to reactivate only a small, optically resolvable fraction of the fluorophores in the field of view at any given time. A typical STORM image was generated from a sequence of ~25–30,000 images acquired at a frame rate of 60 Hz. Super-resolution images were rendered using the fitted coordinates of single molecules by depicting them as 2D Gaussian peaks using the 3D DAOSTORM software package (75).

### Live-cell labeling of F-actin, membranes, and synaptic sites

F-actin was labeled in live cultured neurons by adding 0.5–2 μM SiR-actin (Cytoskeleton, CY-SC001) to the culture medium for 1 h at 37°C, followed by 3 washes in live imaging buffer prior to imaging. Plasma membranes were labeled for live imaging with CellBrite Steady 650 (Biotium, 30108-T), diluted 1:1000 in culture medium with 1X enhancer, incubated for 30 minutes at 37°C, and washed for 15 minutes in live imaging buffer with three exchanges.

Actively-recycling synapses were labeled with SynaptoRed C2M (Biotium, 70019) as follows: cells were habituated for 10 min in HEPES-buffered Tyrode’s solution, consisting of 128 mM NaCl, 5 mM KCl, 2 mM CaCl_2_, 1 mM MgCl_2_, 25 mM HEPES, and 30 mM glucose, supplemented with 5 *μ*M DNQX and 50 *μ*M APV (Sigma) to prevent recurrent excitation. Cells were stimulated with 45 mM KCl for 90 s in buffer containing 10 *μ*M SynaptoRed C2M, followed by 90 s recovery in 5 mM KCl with SynaptoRed. Cells were washed for a total of 15 minutes in live-cell imaging buffer with three exchanges prior to imaging.

### Live cell imaging

For live-cell imaging, coverslips of cultured neurons were transferred to a Chamlide magnetic imaging chamber (CF-T18, Live Cell Instrument/LCI) or were plated in 35 mm MatTek glass-bottom dishes (P35G-1.5-14-C, MatTek). Culture medium was exchanged for live-cell imaging buffer consisting of Hibernate-E Low Fluorescence (TransnetYX) supplemented with 2% B27, 2 mM GlutaMax, 37.5 mM NaCl, and 0.4% w/v D-glucose. Cells were maintained at 37°C during imaging using an Chamlide CU-501 stage-top incubation system, including objective heater (Live Cell Instrument/LCI) or a full-cage environmental chamber on the Zeiss ELYRA7. Oblique-incidence imaging was performed on the custom-built Olympus IX71 microscope used for STORM imaging (see “Super-resolution imaging” section above), acquiring images at 1 s or 15 s intervals with an exposure time of 150 ms. 2D Lattice SIM imaging was performed on a Zeiss ELYRA7 super-resolution microscope, collecting 13 phase images when sampling at 15-sec intervals, and 9 phase images when sampling at 1 s intervals. Photobleaching was minimized by the use of low laser power (typically ~0.4%) and exposure times.

### Quantification of MPS remodeling

Quantification of MPS remodeling from live-cell SIM movies was carried out as follows. We visually identified and marked regions of the axon in which the MPS undergoes at least one degradation and reformation cycle during the first 15 frames of imaging at 15-sec time resolution, corresponding to 225-sec total imaging time, using Fiji/ImageJ. Regions of MPS disassembly were identified by a reduction in the periodic-ring signal (typically by 80-100%) to near-background, while regions of MPS reformation were identified by the reappearance of distinct rings, typically with intensities that were at least 30% of the initial intensity before disassembly. Such regions appeared at different times over the length of the axon, and many such regions undergo 2–3 cycles of degradation and reformation. We then summed the lengths of all regions and report the prevalence of MPS remodeling as the fraction of total axon length undergoing remodeling. At least 10 axons were measured per condition from 2–3 independent replicates for perturbations affecting MPS remodeling.

For quantifying the prevalence of MPS remodeling within or outside synaptic sites, synapses in live, spectrin-mNeonGreen expressing neurons were labeled with SynaptoRed C2M (see “Live-cell labeling of F-actin, membranes, and synaptic sites” section above) prior to two-color SIM imaging to monitor MPS dynamics in regions inside or outside synapses. Sites of MPS remodeling that occurred within bright clusters of SynaptoRed fluorescence are considered synaptic. For comparing MPS dynamics in different regions of the axon, medial–distal regions are defined as the regions of the axon at a distance between 200 and 600 *μ*m from the soma; proximal regions are defined as within 50 *μ*m of the soma.

### Correlation analysis of MPS dynamics and plasma membrane fluctuations

To examine whether MPS remodeling was correlated with bulk flow or other passive movements of the plasma membrane, spectrin-mNeonGreen expressing neurons were labeled with the membrane dye, CellBrite Steady 650, prior to two-color SIM imaging. Regions of the MPS undergoing remodeling were subdivided in time into disassembly events and reassembly events (transitions). For each event, the fluorescence intensities of the MPS (spectrin-mNeonGreen signal) and the membrane (CellBrite signal) in transitioning regions were measured over time. Correlation between the two signals was examined by plotting the fluorescence intensities of the MPS and membrane before and after each transition, or by generating a scatter plot of MPS intensities versus membrane intensities and determining the Pearson correlation coefficient between the two signals.

### Fluorescence autocorrelation analysis of diffraction-limited movies and screening

Quantification of spectrin dynamics in diffraction-limited movies was performed using fluorescence autocorrelation analysis (76). Kymographs were extracted from individual axons in each movie, and the Pearson linear correlation ⍴ between each pair of kymograph columns separated by time interval Δt up to a maximum Δt of 150 s was computed. The autocorrelation function value at Δt is calculated as the median of ρ for all pairs of kymograph columns separated by Δt:r(Δt)=median(ρ(Kymo(t),Kymo(t+Δt))). This autocorrelation function was fit with a single exponential of the form $ae^{-k\Delta t}+b$, where the amplitude, a, was used to quantify the prevalence of MPS remodeling. For screening potential regulators of MPS remodeling, neurons were treated with pharmacological inhibitors at the concentrations and durations given in the Table S1 or with shRNAs, and the average autocorrelation amplitudes from 20–30 axons pooled from 2–3 independent experimental replicates were compared with controls. Perturbations yielding significant fold changes over controls were selected for further analysis by live-cell SIM imaging.

### Pharmacological perturbations

Neurons at DIV14–16 were treated with the drugs as indicated in Table S1, along with solvent control (typically DMSO), maintained at the same concentration in imaging media during imaging.

### Calcium reporter dyes

To measure Ca^2+^ spiking activity in cultured neurons, cells were loaded with the Ca^2+^ indicator dye Cal-520 (AAT Bioquest, 21130) at a concentration of 2 *μ*M in cell culture media for 45 minutes prior to live-cell TIRF imaging. Cells were imaged at a rate of 2 Hz with an exposure time of 100 ms, and spiking activity was assayed by plotting normalized changes in fluorescence intensity according to the equation $F(t)=\left(F(t)-F_{o}\right)∕F_{o}$, where F(t) is the fluorescence intensity at time t and $F_{o}$ is the initial intensity. Resting Ca^2+^ concentrations were estimated by loading cells with the ratiometric dye, CalRed R525/650, according to the same protocol, where the ratio of emission at 525 nm to 650 nm indicates the relative Ca^2+^ concentration and allows a normalization for differences in dye loading between experimental conditions. For comparing the relative resting Ca^2+^ concentration in cells treated with BAPTA, TTX, nimodipine, ω-conotoxin, or control, 5–12 fields of view (FOVs) of non-spiking neurites were collected, and the average fluorescence ratio of all neurites per FOV were plotted.

### Calcium uncaging

Ca^2+^ uncaging experiments were performed by loading neurons with 10–30 *μ*M *o*-Nitrophenyl EGTA AM (NP-EGTA, ThermoFisher) for 45 min in cell culture media prior to transfer to live-cell imaging media. Uncaging was performed by delivering a 6-sec pulse of intense 405-nm laser illumination to small regions of axons, or by continuously delivering low-intensity illumination, while simultaneously imaging fluorescent protein constructs or dyes. The power and duration of 405 nm illumination to induce uncaging was determined empirically by imaging neurons loaded with the Ca^2+^ indicator dye Cal-520 (AAT Bioquest, 21130) as above and varying the intensity and duration of 405 nm laser pulses. Small axonal regions were targeted by inserting an iris mounted in a motorized filter wheel (FW102C, Thorlabs) into the excitation path. Ca^2+^ uncaging over large regions of coverslips was achieved by delivering pulses 405 nm illumination to the entire FOV, and automating tiled image acquisition over a 10 × 10 grid, covering an area of 400 × 400 *μ*m in ~5 minutes. The timing of laser pulses and the iris during image acquisition were controlled by custom microscope control software and data acquisition card (National Instruments, PCIe-6321). Ca^2+^ uncaging during live-cell SIM imaging was achieved by continuous low-power 405 nm excitation during imaging.

### Characterization of MPS imperfections in fixed imaging

To examine the correspondence between the prevalence of MPS remodeling observed in live-cell imaging and the appearance of the MPS in fixed-cell imaging, we trained a fully convolutional neural network to classify regions of the MPS as being “imperfect” or “intact” in SIM images of fixed neurons using the STEDActinFCN software package (34). The training set supplied to the software consisted of seven 2D SIM images of *β*II spectrin in axons of DIV 14 neurons, in which regions of imperfect and intact MPS were manually annotated. Following verification of the software’s model to accurately classify regions of the MPS by visual inspection, 27 large FOV SIM images were supplied, yielding a set of 66 axons in which regions of the MPS were segmented by their classification. The prevalence of imperfect MPS in axons was quantified as a fraction of the total area covered by the axon.

### Correlation analysis of spatial distributions in super-resolution imaging

One-dimensional (1D) autocorrelation analyses along the length of axons were used to measure the periodicity of the MPS and associated proteins in regions of axons, as previously described (10). For SIM images, regions were chosen at random from 10–60 *μ*m stretches of axons, and a 1D pixel intensity profile along the long axis of the axons was used; for STORM images, regions were chosen from 2–5 *μ*m stretches, and the localization distribution was projected along the axonal long axis to obtain a 1D distribution profile. The 1D autocorrelation function, AC(Δx) was calculated from these profiles using the equation:

$$AC(\Delta x)=\frac{\sum_{x=1}^{N-\Delta x}(I(x)-\bar{I})(I(x+\Delta x)-\bar{I})}{\sum_{x=1}^{N}{\left(I(x)-\bar{I}\right)}^{2}},$$

where $\bar{I}$ is the average of I(x), the 1D spatial distribution of the signal, and Δx is the lag. The average 1D autocorrelation function was determined from 50–100 axonal regions unless otherwise indicated. For quantifications of spatial autocorrelation amplitude, the amplitude was defined as the difference between the first peak (located at ~190 nm when considering only positive values) and the average of the first two valleys (at approximately ~95 nm and ~285 nm) of the average autocorrelation function.

To compare the spatial distributions of proteins relative to spectrin in two-color SIM images, we obtained 1D intensity profiles as above and computed the 1D cross-correlation function CC(Δx) using the equation:

$$CC(\Delta x)=\frac{\sum_{x=1}^{N-\Delta x}(I_{1}(x)-\bar{I_{1}})(I_{2}(x+\Delta x)-\bar{I_{2}})}{\sum_{x=1}^{N}(I_{1}(x)-\bar{I_{1}})(I_{2}(x)-\bar{I_{2}})},$$

where $I_{1}(x)$ and $I_{2}(x)$ are the 1D spatial distributions of the two channels, $\bar{I_{1}}$ and $\bar{I_{2}}$ are their averages, and Δx is the lag.

### Correlation of calpain activity with MPS degradation

To examine the correlation between calpain activity and MPS degradation, we used an antibody specific to *α*II-spectrin that has been cleaved by calpain as a reporter of calpain activity in fixed and immunostained neurons. Calpain activity was quantified by measuring the fluorescence intensity of cleaved *α*II-spectrin staining, and the degree of MPS degradation was quantified from super-resolution images by measuring the spatial autocorrelation amplitude of *β*II-spectrin in the same regions of the axon. To generate the correlation plot in Fig. 4D, regions of immunostained axons with varying cleaved spectrin intensities were grouped into 12 equally-spaced bins and the fraction of regions with degraded MPS was plotted. Regions of the MPS exhibiting an autocorrelation amplitude less than 0.2 (see “Correlation analysis of spatial distributions in super-resolution imaging”) were classified as having degraded MPS, confirmed by visual inspection.

### Endocytosis assays

To measure endocytosis in axons, unstimulated neurons were incubated with either 5 *μ*g/mL low-density lipoprotein (LDL) labeled with Alexa Fluor 594 (ThermoFisher, L35353) for 90 minutes or 4 *μ*M of the lipophilic dye, AM4-65 (Biotium, 70039), for 30 minutes at 37°C. Cells were washed for 15 minutes in ice-cold Tyrode’s solution containing 0.5 *μ*M TTX with three exchanges, followed by fixation, immunostaining, and imaging by widefield or confocal microscopy. In drug perturbation experiments, cells were treated with drugs for 30 minutes prior to the endocytosis assay. LDL endocytosis was quantified as the number of fluorescent puncta, corresponding to internalized LDL, per unit area of the axon and normalized to DMSO-treated control. Fluorescent puncta in axons were counted by manually segmenting axons in Fiji/ImageJ on the basis of a membrane co-stain, followed by automated spot detection using the ComDet ImageJ plugin (https://github.com/UU-cellbiology/ComDet) with an approximate particle diameter of 300 nm and an intensity threshold of 25 standard deviations above background. AM dye endocytosis was quantified by measuring the internalized dye fluorescence following manual segmentation of axons using a membrane co-stain as above.

### Statistics

All statistical analyses and plotting were performed in the R statistical environment (77). In boxplots, the horizontal line depicts the median and the box depicts the interquartile range (IQR) of the data. Whiskers extend to 1.5 times the IQR, with potential outliers omitted. Bar plots depict the mean ± SEM. A two-tailed, unpaired t-test was used to assess the level of significance between two experimental conditions. When three or more conditions were compared, we used a one-way or two-way ANOVA followed by Tukey’s Honestly Significant Difference (HSD) post-hoc test. An F-test was used to determine the significance of a fit when using linear regression. For assessing statistical significance when comparing time-series data, the mean of the final three timepoints was used. In figures, significance is indicated as follows: n.s., not significant; *, *p* < 0.05; **, *p* < 0.01; ***, *p* < 0.001. A complete description of *p* values, replicate numbers, and statistical tests used for the results presented in individual figures is provided in Table S2.

## Supplementary Material

Movie S1

Supplementary Materials

Figs. S1 to S16

Tables S1 and S2, Movie S1
