# Supplementary material for: The membrane skeleton is constitutively remodeled in neurons by calcium signaling
Source: Science. Author manuscript; Available in PMC 2025 Aug 8. (PMC12333566; doi:10.1126/science.adn6712)
Supplement: Supplementary Materials [file NIHMS2098478-supplement-Supplementary_Materials.pdf]

Science
AAAS

Supplementary materials for

The membrane skeleton is constitutively remodeled in neurons by calcium signaling

Evan Heller, Naina Kurup, Xiaowei Zhuang

Correspondence to: zhuang@chemistry.harvard.edu (X.Z.)

This PDF includes:

Figs. S1 to S16

Tables S1 and S2

Movie S1 caption

Other Supplementary Materials for this manuscript include the following:

Movie S1

Supplementary figures

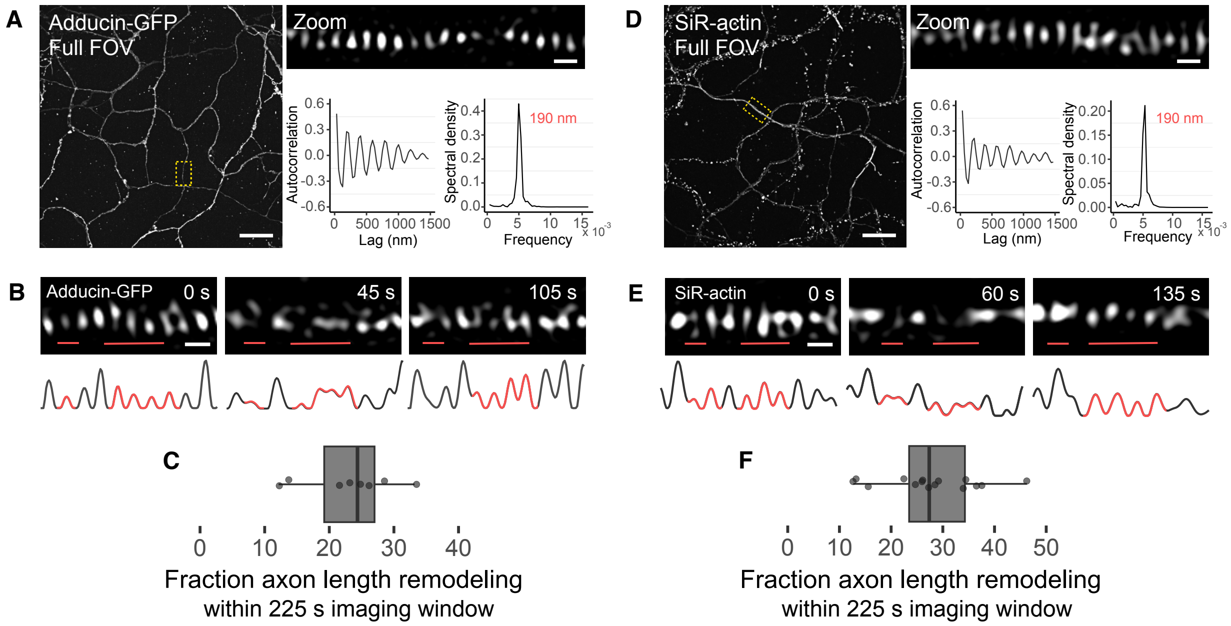

Fig. S1. Live-cell SIM imaging of alternative MPS markers. (**A**) Live-cell SIM image of a DIV 15 neuron expressing adducin-GFP. *Left,* full FOV. Right top: zoom-in of boxed region on the left. Right bottom: autocorrelation function of the adducin-GFP signal along the axon (left, averaged over 34 axonal regions) and spectral density analysis of the autocorrelation function (right). (**B**) Example MPS remodeling from live-cell SIM imaging of adducin-GFP neurons acquired at a 15-sec time resolution during a standardized 225-sec imaging window, in which regions of the MPS undergo cycles of disassembly and reassembly. Dynamic regions in each frame are underlined in red, with intensity traces illustrating loss and reformation of the MPS shown below. (**C**) Quantification of the prevalence of MPS remodeling (fraction of axon length undergoing remodeling during the 225-sec imaging window) using adducin-GFP as a label. (**D**) Live-cell SIM imaging of DIV 15 neurons labeled with SiR-actin and characterization of the MPS as in (A). An average of 29 axonal regions is plotted in the autocorrelation analysis. (**E**) Example MPS remodeling as in (B) using SiR-actin as a label. (**F**) Quantification of the prevalence of MPS remodeling as in (C) using SiR-actin as a label. Statistics for (C, F) are shown in Table S2. Scale bars, 10 μm (A, D); 250 nm (A, zoom; B; D, Zoom; E).

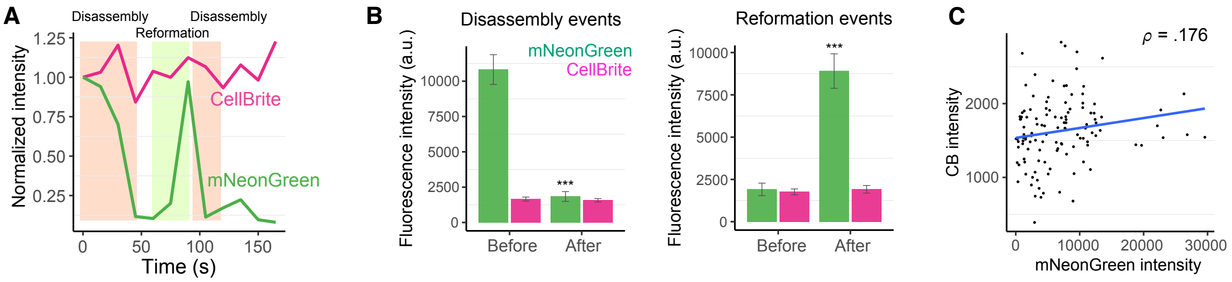

Fig. S2. SIM imaging of MPS remodeling controlled with imaging of the plasma membrane marker. (**A**) Representative fluorescence intensity traces of spectrin-mNeonGreen and the plasma membrane stain, CellBrite Steady, in an axonal region undergoing MPS disassembly (red shaded areas) and reassembly (green shaded areas) from live, two-color SIM imaging. The spectrin-mNeonGreen signal undergoes large transitions, whereas the CellBrite signal remains largely constant. (**B**) *Left*, quantification of the mean spectrin-mNeonGreen and CellBrite Steady intensities before and after MPS disassembly events. The mNeonGreen signal exhibits an ~6-fold decrease in intensity during MPS disassembly, whereas the CellBrite signal is virtually unchanged. *Right*, corresponding quantification of MPS reassembly events. The mNeonGreen signal exhibits an ~5-fold increase in intensity during reassembly, whereas the CellBrite signal is unchanged. (**C**) Spectrin-mNeonGreen and CellBrite fluorescence intensity are poorly correlated as MPS transitions take place. Axonal regions undergoing MPS disassembly and reassembly were selected from live, two-color SIM movies. For each disassembly or reassembly transition, timepoints immediately before, during, and immediately after the transition were collected and spectrin-mNeonGreen intensity was plotted against CellBrite intensity for each time point. The low value of the Pearson correlation coefficient indicates a poor correlation of the two signals, supporting the notion that MPS remodeling occurs independently of passive fluctuations of the membrane. A total of 240 transitions are included in the plot. Statistics for (B, C) are shown in Table S2.

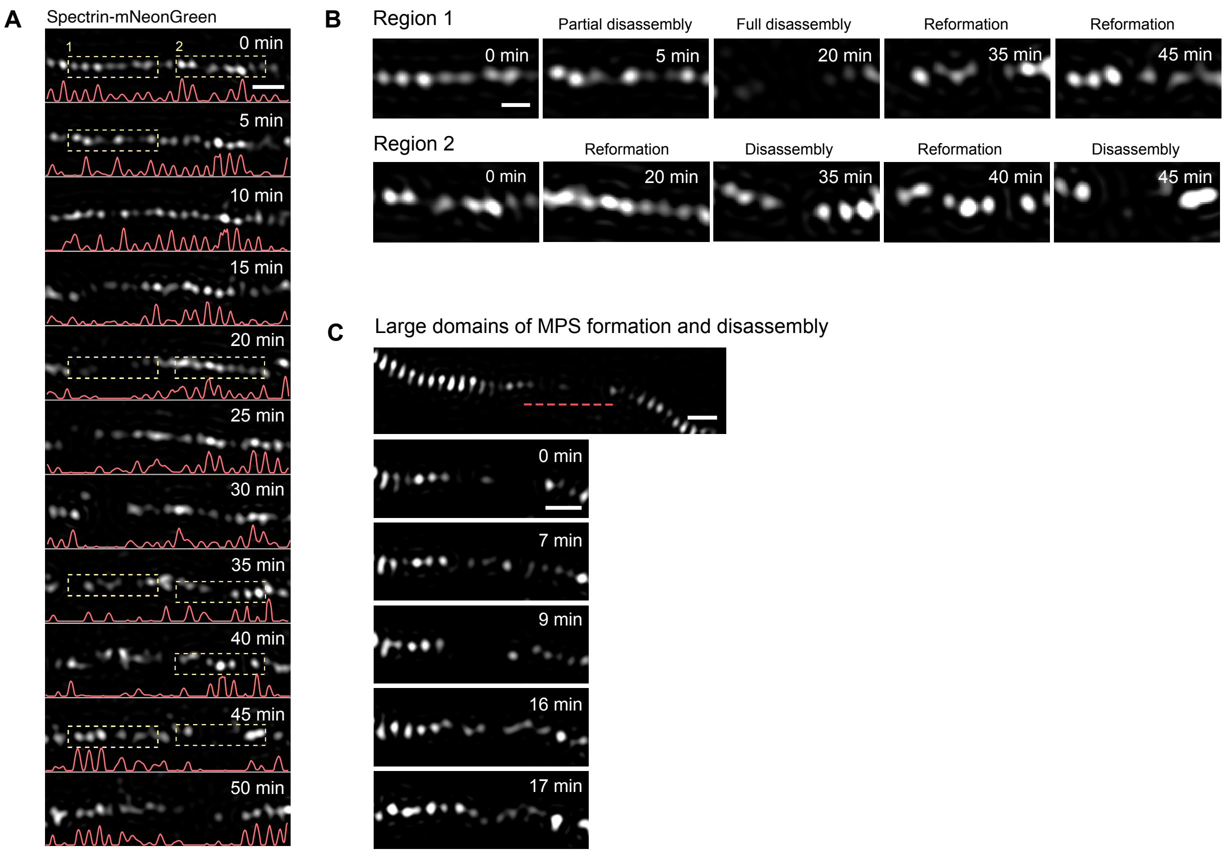

Fig. S3. Remodeling of the MPS observed at long time scales. (**A**) Full sequence of live-cell SIM imaging of the MPS acquired at a 5-min time resolution, in which large stretches of the MPS undergo cycles of degradation and reformation over the course of 1 hour. In each frame, intensity traces are shown in red. (**B**) Annotated, close-up views of the regions indicated in (A), in which large stretches of the MPS undergo cycles of degradation and reformation. (**C**) Additional example of MPS degradation and reformation in a large stretch of an axon (red dashed line). Many axons, particularly in medial–distal regions, contain stretches in which the MPS is relatively sparse or absent. In live-cell SIM imaging, these regions are observed to undergo bouts of formation and degradation. Scale bars, 500 nm (A, C); 250 nm (B).

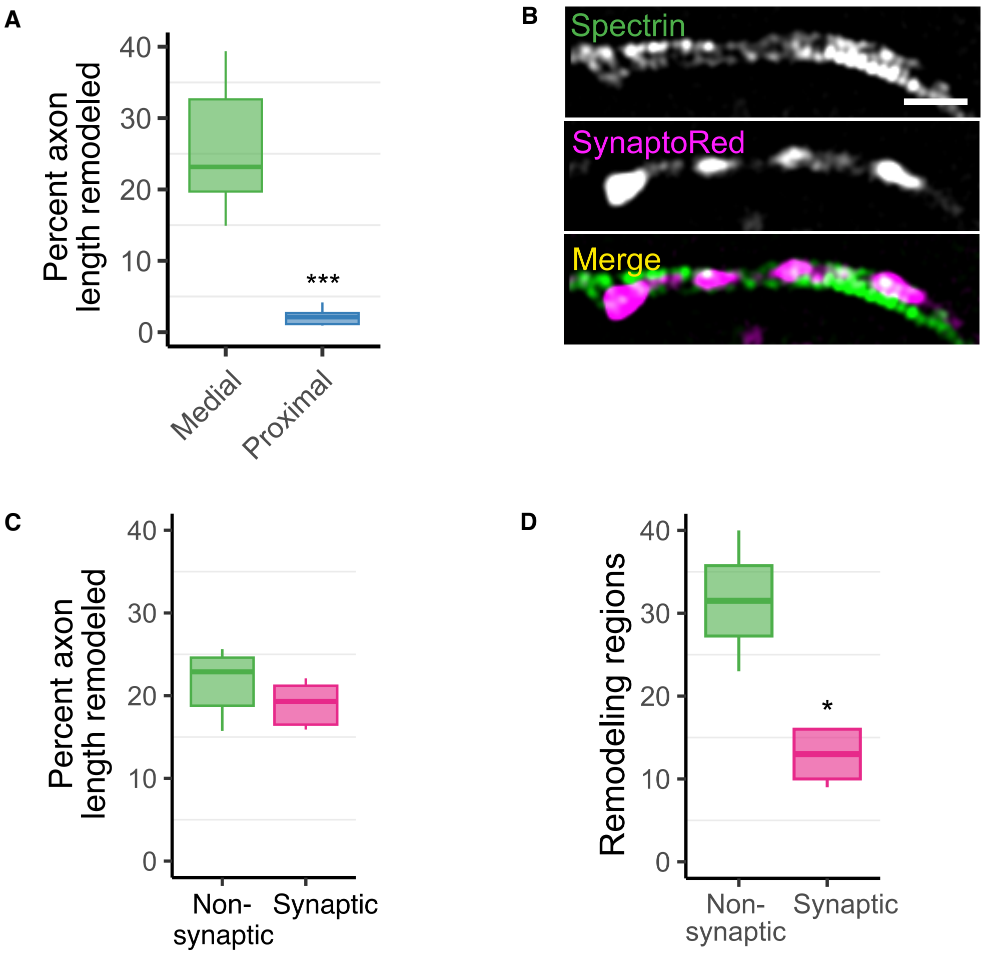

Fig. S4. MPS remodeling primarily occurs in medial-distal axons in both synaptic and non-synaptic sites. (**A**) Prevalence of MPS remodeling (fraction axon length undergoing remodeling over the course of 225-sec of imaging) in medial-distal axons (typically between 200 μm and 600 μm of the soma) and proximal axons (within 50 μm of the soma). (**B**) Representative snapshots from live-cell two-color SIM imaging of spectrin-nNeonGreen (green) and the synaptic marker, SynaptoRed C2M (magenta), in an axon. Scale bar, 1 μm. (**C**) Quantification of the fraction of axon length exhibiting MPS remodeling within and outside of synaptic regions. Synaptic sites are defined as regions of the axon within a bright cluster of SynaptoRed intensity. (**D**) Quantification of the number of remodeling regions in synaptic and non-synaptic sites per axon, indicating that the majority of MPS remodeling occurs outside of synaptic sites. Statistics for (A, C, and D) are shown in Table S2.

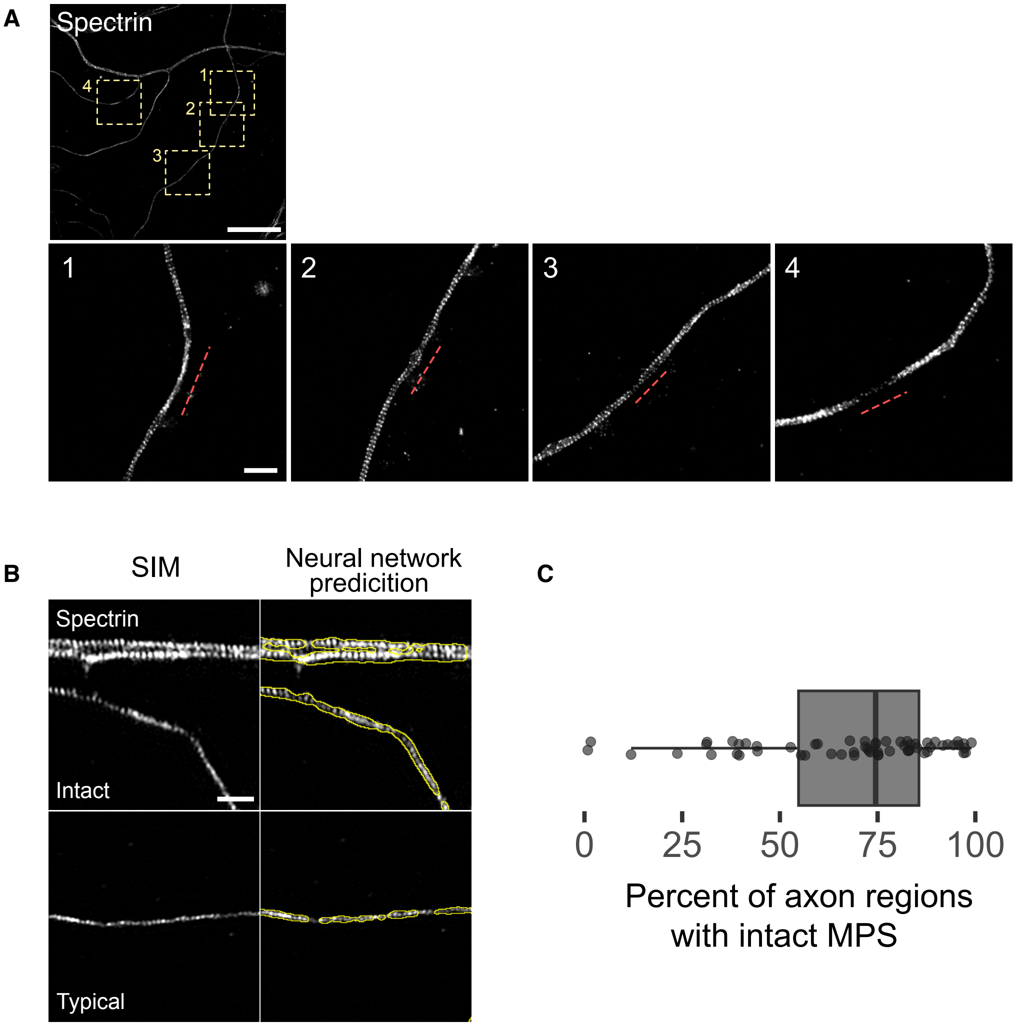

Fig. S5. Characterization of MPS integrity in fixed-cell imaging. (**A**) *Top*, example large-FOV STORM image of immunolabeled spectrin in fixed DIV 15 rat neurons. *Bottom*, zoom-in views of the boxed regions above. The MPS exhibits regions of local imperfections (dashed red lines). Scale bars, 10 μm (top), 1 μm (bottom). (**B**) Training of a fully convolutional neural network using the `STEDActinFCN` software package (*34*) to classify regions of the MPS as being intact or imperfect from super-resolution images (see *Materials and Methods*). *Left panels*, example images of regions with mostly intact MPS, more common in proximal axons, as well as more typical regions of the MPS in medial axons, which contains subregions showing imperfections. *Right panels*, output of the neural network. The neuronal network segments axons into regions with intact MPS (yellow outlines) and imperfect MPS (without outlines). Scale bar, 1 μm. (**C**) Quantification of the fraction of axonal regions exhibiting intact MPS from the output of the neural network. Statistics for (C) are shown in Table S2.

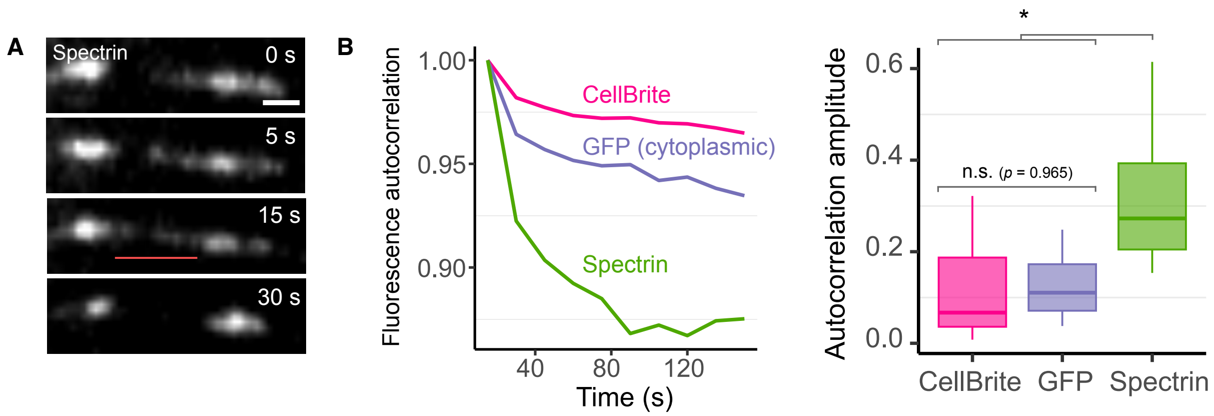

Fig. S6. Diffraction-limited imaging of spectrin for screening regulators of MPS remodeling. (**A**) Representative snapshots from diffraction-limited live-cell imaging of spectrin-mNeonGreen in DIV 15 neurons. Focusing on a region where cycles of appearance and disappearance of the spectrin signal are observed (marked by red line), similar to the degradation and reformation of MPS rings seen by live-cell SIM. Images acquired at 1-sec intervals. Scale bar, 1 μm. (**B**) Fluorescence autocorrelation analysis of spectrin-mNeonGreen (green), a cytoplasmic marker (GFP, purple), and a membrane marker (CellBrite, pink). Autocorrelation function is determined as described in *Materials and Methods* for each molecular marker. The median autocorrelation is plotted on the left, while autocorrelation amplitude distributions derived from individual axons are plotted on the right. Statistics for (B) are shown in Table S2.

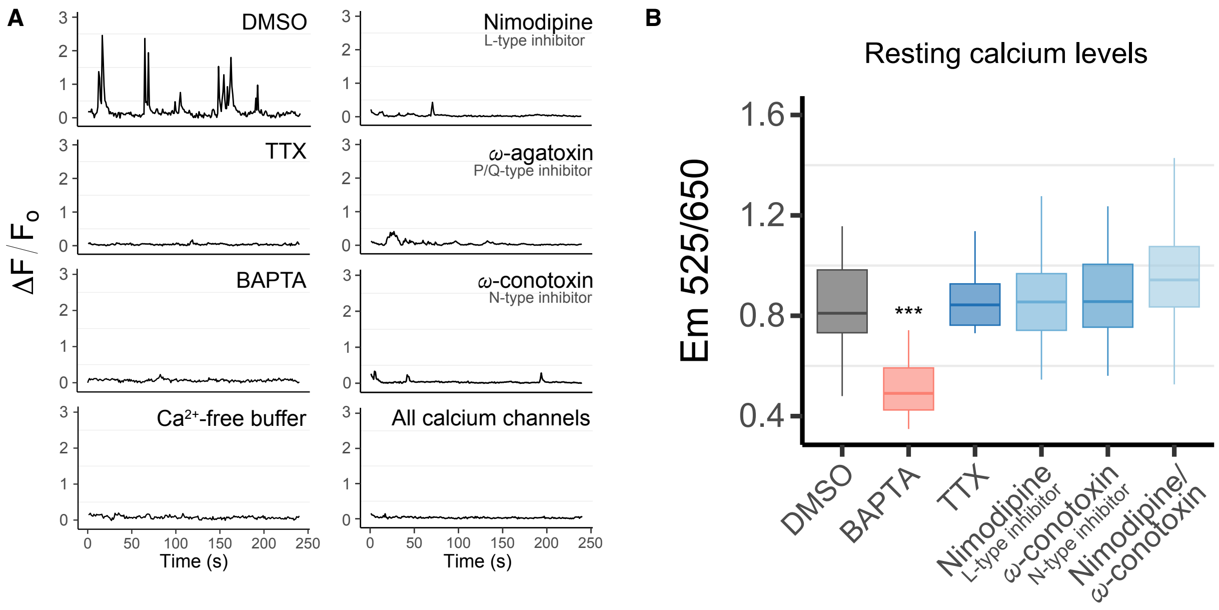

Fig. S7. Effect of various perturbations to Ca^{2+}, Ca^{2+} channels, and action potentials on Ca^{2+} spiking and resting levels. (**A**) Representative plots of spontaneous neuronal activity in DIV 14 rat neurons measured using the Ca^{2+} reporter dye, Cal-520, in the presence of DMSO (control), TTX (an action potential inhibitor), BAPTA (a Ca^{2+} chelator), nimodipine (a L-type Ca^{2+} channel inhibitor), ω-agatoxin IVA (a P/Q-type Ca^{2+} channel inhibitor), ω-conotoxin (a N-type Ca^{2+} channel inhibitor), or all three Ca^{2+} channel inhibitors, as well as in a Ca^{2+}-free buffer. Normalized changes in fluorescence intensity, $\Delta F/F_o$, are plotted to visualize spiking amplitude and frequency. $\Delta F/F_o = (F(t) - F_o)/F_o$, where $F(t)$ is the fluorescence intensity at time t and F_o is the initial intensity. Neurons exhibit low-amplitude, asynchronous Ca^{2+} spikes as well as periodic bursts of synchronized activity. This spiking activity is completely inhibited by application of TTX, BAPTA, or calcium-free buffer (left panels). Inhibition of voltage-gated Ca^{2+} channels, including L-type channels (with nimodipine), P/Q-type channels (with ω-agatoxin), and N-type channels (with ω-conotoxin) similarly inhibit the majority of spiking activity, preserving only infrequent, low-amplitude residual activity (right panels). (**B**) Quantification of resting Ca^{2+} levels in cultured neurons using the ratiometric dye, CalRed R525/650. A higher ratio of emission at 525 nm to 650 nm indicates a higher Ca^{2+} concentration. Chelation of Ca^{2+} with BAPTA strongly reduces resting Ca^{2+} levels, whereas application of TTX or the inhibition of L-type and N-type Ca^{2+} channels have little effect. Statistics for (B) are shown in Table S2.

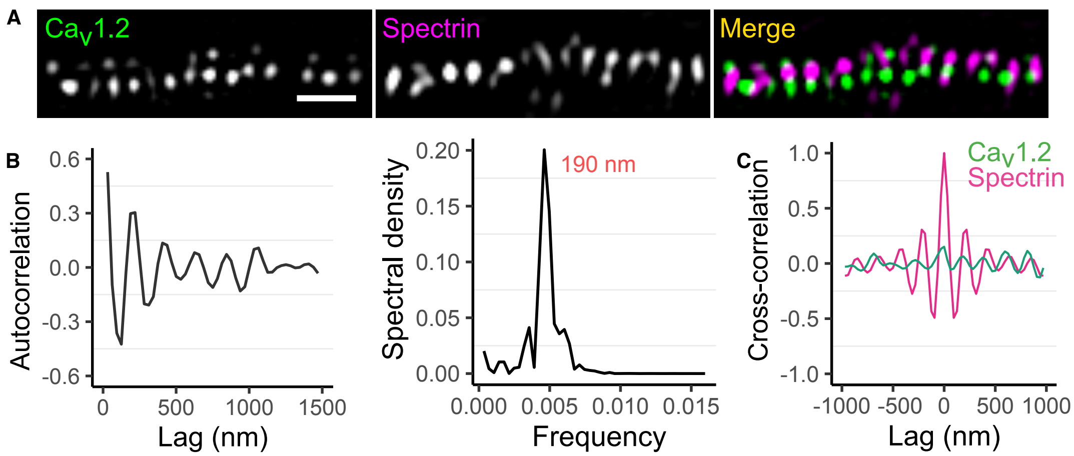

Fig. S8. Ca^{2+} channels are partially associated with the MPS. (**A**) Representative two-color SIM imaging of the L-type Ca^{2+} channel, $Ca_v1.2$, and spectrin in fixed neurons, illustrating the periodic distribution and colocalization of $Ca_v1.2$ with spectrin in some regions of axons, indicating transient or local association with the MPS. Scale bar, 500 nm. (**B**) *Left*, average autocorrelation of 10 axonal regions exhibiting the highest $Ca_v1.2$ periodicity by visual inspection. *Right*, spectral analysis of 46 axonal regions from 4 FOVs shows a peak at 190 nm, indicating a periodic distribution matching that of spectrin. (**C**) Cross-correlation analysis of $Ca_v1.2$ (green) and spectrin (magenta) for 10 axonal regions exhibiting the highest $Ca_v1.2$ periodicity by visual inspection.

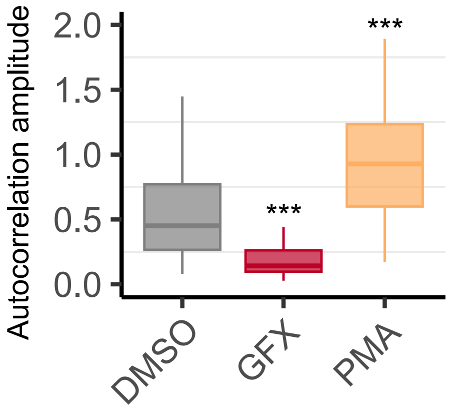

Fig. S9. PKC activity promotes MPS dynamics. Autocorrelation amplitudes derived from diffraction-limited imaging of spectrin-mNeonGreen in axons treated with the PKC inhibitor, GFX (10 μM), the PKC activator, PMA (1 μM), or DMSO control 30 min prior to imaging. PKC inhibition reduces MPS dynamics, whereas PKC activation increases MPS dynamics. Statistics are shown in Table S2.

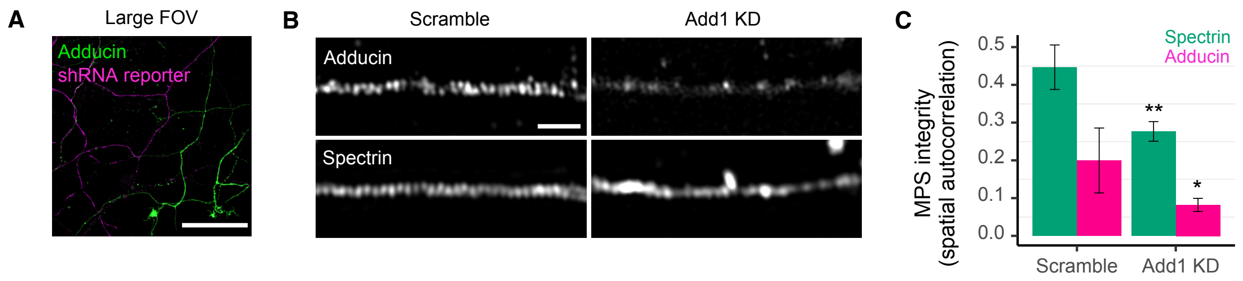

Fig. S10. Validation of adducin shRNA. (**A**) Large-FOV SIM image of adducin immunostaining (green) with mosaic expression of virally delivered adducin shRNA (magenta) in DIV 15 neurons, illustrating a specific loss of adducin following knockdown. Cells were treated at a multiplicity of infection (MOI) of 25 for 5 days prior to fixation and imaging. (**B**) Zoom-in images of axons in control shRNA-treated and adducin shRNA-treated neurons stained for both adducin (top) and spectrin (bottom). A partial depletion of adducin moderately affected the integrity of the MPS. (**C**) Quantification of MPS integrity in neurons treated with control shRNA or adducin shRNA. The average spatial autocorrelation amplitude, reflecting the degree of periodicity of the MPS, was calculated for spectrin (green) and adducin (magenta). Statistics for (C) are shown in Table S2. Scale bars, 10 μm (A), 1 μm (B).

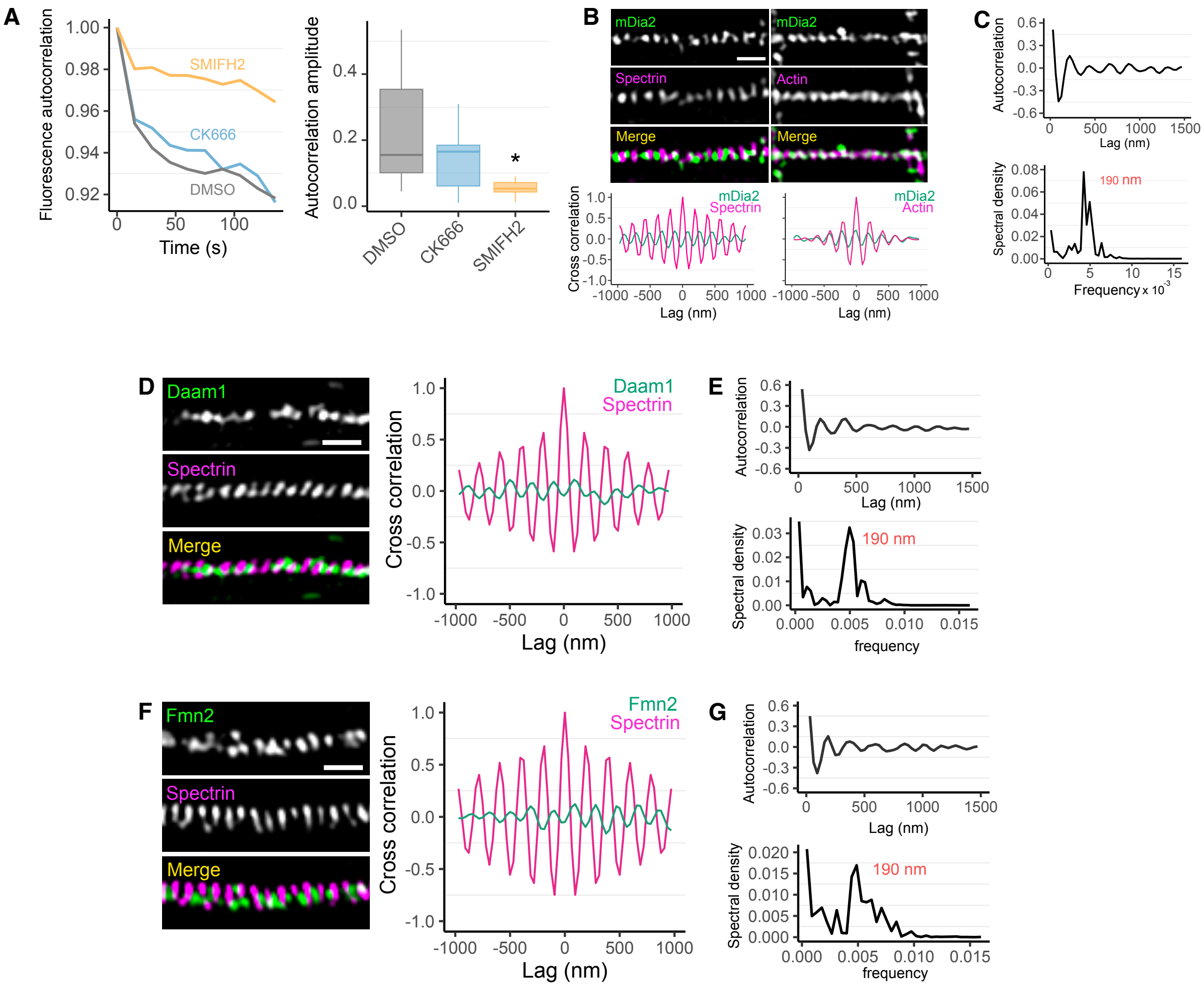

Fig. S11. Formins are partially associated with the MPS and contribute to dynamics. (**A**) Median fluorescence autocorrelation curves (left) and autocorrelation amplitudes (right) derived from diffraction-limited imaging of spectrin-mNeonGreen in axons treated with the Arp2/3 inhibitor, CK666 (15 μM, 30 min prior to imaging), or the formin inhibitor, SMIFH2 (30 μM, 10 min prior to imaging), in comparison to DMSO control. Statistics are shown in Table S2. (**B–G**) Formins are associated with actin rings in isolated stretches of the MPS, indicating transient or local association. (**B**) *Top*, representative two-color SIM images of the formin, mDia2 (green), and spectrin (magenta, left) or actin (right), illustrating the periodic distribution of mDia2 in an alternating pattern to that of spectrin and overlapping with that of actin in some regions of axons. *Bottom*, cross-correlation analysis of mDia2 (green) and spectrin (magenta, left) or actin (magenta, right) distributions in two-color SIM images from 6 regions exhibiting the highest mDia2 periodicity by visual inspection, suggesting an association of mDia2 with actin rings. (**C**) Autocorrelation analysis of the mDia2 signal in axonal regions exhibiting periodic distributions. The average autocorrelation of 28 axonal regions exhibiting periodic distributions by visual inspection (top) with corresponding spectral density analysis (bottom) indicating a periodicity of ~190 nm matching that of spectrin. (**D**) *Left*, representative two-color SIM images of the formin, Daam1 (green), and spectrin (magenta) as in (B). *Right*, cross-correlation analysis of Daam1 (green) and spectrin (magenta). (**E**) Autocorrelation analysis of Daam1 distribution axons as in (C). (**F–G**) As in (D-E) but for formin-2 (Fmn2). Scale bars, 500 nm.

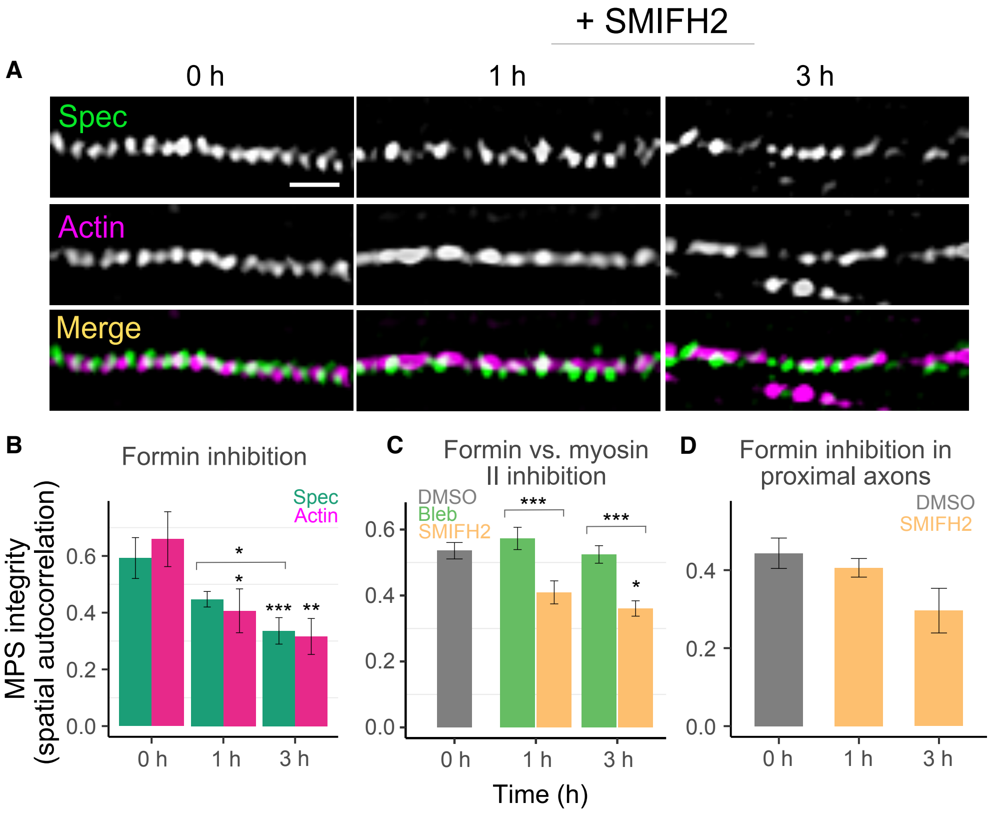

Fig. S12. Role of formin activity in MPS maintenance. (**A**) DIV 15 neurons were treated with 10 μM of the formin inhibitor, SMIFH2, for the indicated durations, followed by fixation, immunostaining for βII-spectrin and staining of F-actin, and SIM imaging. Scale bar, 500 nm. (**B**) Quantification of MPS integrity following inhibition of formins with SMIFH2. The average autocorrelation amplitude is plotted for spectrin (green) and actin (magenta), illustrating a decreased average autocorrelation amplitude for both. (**C**) Comparison of the effect of formin inhibition to inhibition of myosin II in axons. Neurons were treated with 10 μM of the formin inhibitor, SMIFH2 (orange), or 10 μM of the myosin II inhibitor blebbistatin (Bleb, green) for the indicated durations, followed by fixation and immunostaining for spectrin. Inhibition of myosin II has little effect on MPS integrity compared to that of formin inhibition, ruling out a non-specific effect of the formin inhibitor, SMIFH2, on myosin II activity in these conditions. (**D**) Quantification of MPS integrity in axon regions proximal to the cell body following inhibition of formins with SMIFH2. Autocorrelation amplitude is plotted from different regions of the same samples analyzed in (C). In comparison to medial-distal axons (B–C), long-term formin inhibition has a relatively weak effect on MPS integrity in proximal axons. Proximal axon regions are defined as regions of the axon with 50 μm of the soma. Statistics in (B-D) are shown in Table S2.

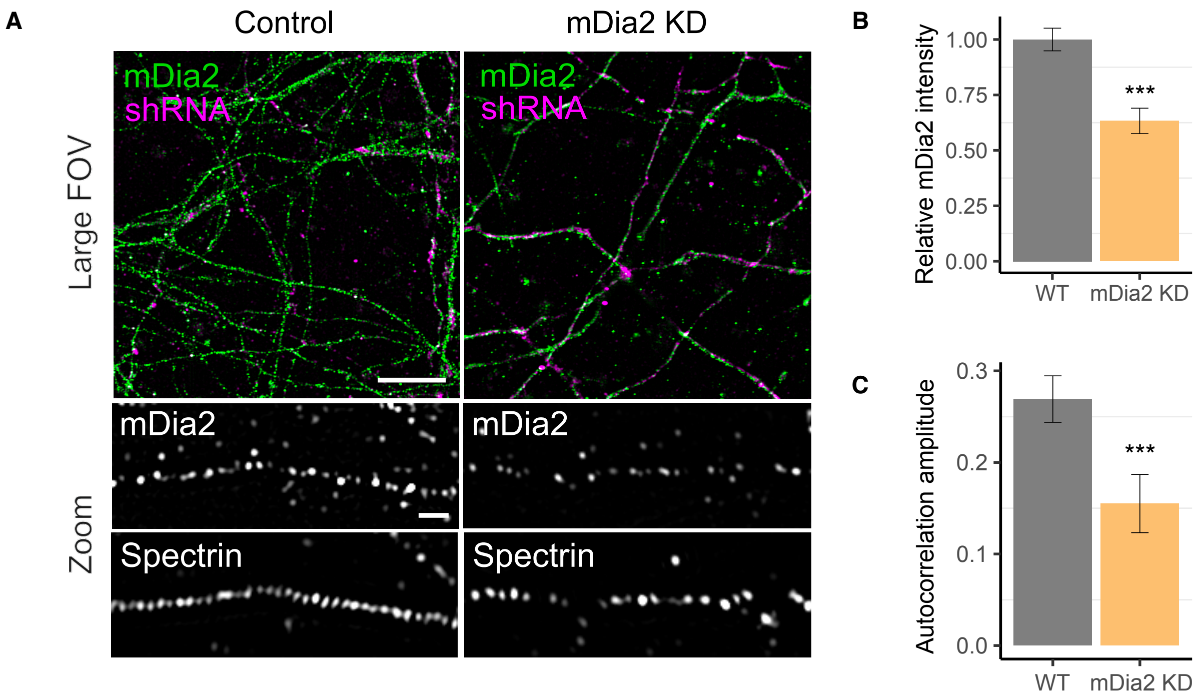

Fig. S13. Knockdown of mDia2 mimics the effect of long-term pharmacological inhibition of formins on MPS integrity. (**A**) *Top*, large-FOV SIM image of mDia2 immunostaining (green) with mosaic expression of mDia shRNA (magenta) in DIV 15 neurons, illustrating a specific loss of mDia2 following knockdown. Cells were transduced with virus carrying mDia shRNA at a MOI of ~25 for 5 days prior to fixation and imaging. *Bottom*, zoom-in images of uninfected axons (left) and mDia2 shRNA-treated axons (right) stained for both mDia2 (top) and spectrin (bottom). (**B**) Quantification of the efficiency of mDia2 knockdown. Following expression of mDia2 shRNA for 5 days, neurons were fixed and immunostained for mDia2, and the fluorescence intensity was quantified from SIM images. (**C**) Quantification of MPS integrity in neurons treated with mDia2 shRNA (orange) compared to uninfected axons within the same sample (gray). The average spatial autocorrelation amplitude, reflecting the degree of periodicity of the MPS, was calculated for spectrin in mDia2 knockdown regions and uninfected control regions in fixed SIM images of the same samples analyzed in (B). Loss of mDia2 results in a significant disruption of MPS integrity. Statistics in (B, C) are shown in Table S2. Scale bars, 10 μm (A, large FOV), 500 nm (A, zoom).

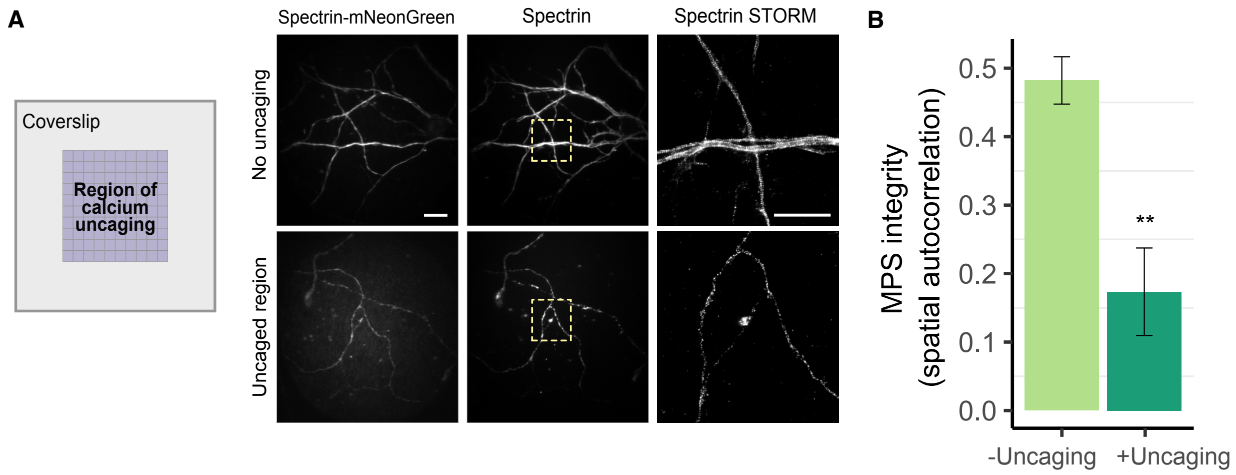

Fig. S14. Ca^{2+} uncaging leads to spectrin degradation. (**A**) Ca^{2+} uncaging leads to spectrin degradation, measured by immunostaining and STORM imaging. Live spectrin-mNeonGreen expressing neurons were treated with 30 μM NP-EGTA (caged Ca^{2+}). Ca^{2+} uncaging was performed in a large central region (400 × 400 μm) of the coverslip by delivering an intense, 6-sec pulse of high intensity 405 nm illumination (schematic on left). Samples were then fixed, immunostained for βII-spectrin, and imaged by STORM. *Top*, conventional images of spectrin-mNeonGreen (left) and immunostained βII-spectrin (middle) in a region not subject to calcium uncaging. STORM image of βII-spectrin in the boxed region is shown on the right. *Bottom*, as in the top panels, but for a region subjected to Ca^{2+} uncaging, showing degradation of the MPS upon Ca^{2+} uncaging. (**B**) Quantification of MPS integrity in axons subject to Ca^{2+} uncaging as compared to control. The average autocorrelation amplitude of the MPS was calculated in regions of the sample subject to uncaging and compared to that in regions without uncaging, showing that Ca^{2+} uncaging leads to a significant disruption of the MPS. Statistics in (B) are shown in Table S2. Scale bars in (A), 5 μm.

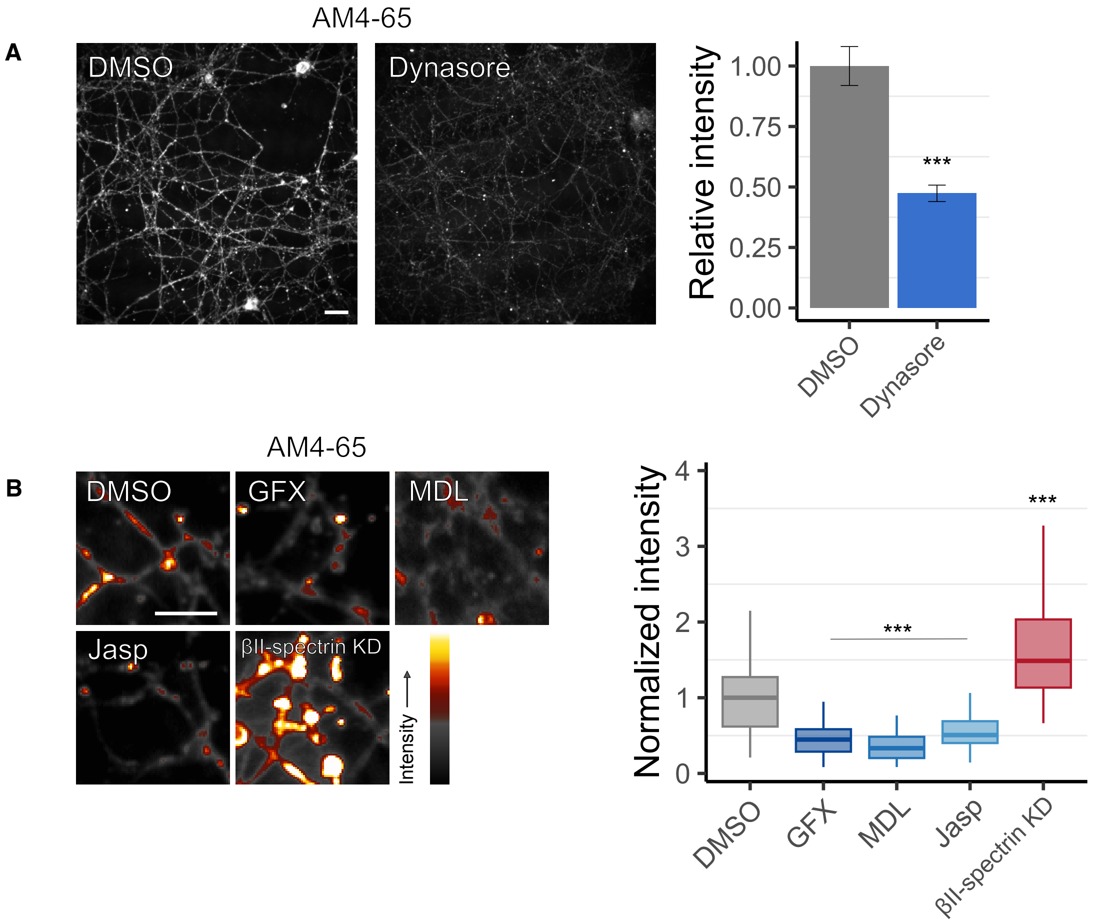

Fig. S15. Endocytosis of lipid membrane following perturbation of MPS dynamics. (**A**) *Left*, representative images of the lipophilic dye, AM4-65, following a 30-min incubation in neurons treated with DMSO (left) and 80 μm dynasore (an inhibitor of clathrin-mediated endocytosis, right) for 30 min prior to AM dye uptake. *Right*, quantification of AM4-65 uptake in DMSO- and dynasore-treated neurons following fixation and imaging by confocal microscopy. Some residual staining remains after treatment with dynasore, either due to incomplete washout of the dye from the plasma membrane or from the contribution of non-clathrin-mediated endocytosis. (**B**) *Left*, representative images of AM dye uptake in live neurons following perturbations that stabilize the MPS and decrease MPS dynamics (PKC inhibition with 10 μM GFX, calpain inhibition with 50 μM MDL, and actin stabilization with 10 μM Jasp) or a perturbation that removes the MPS (βII-spectrin knockdown). Perturbations that inhibit MPS dynamics all result in a significant decrease in endocytosis, whereas removal of the MPS causes an increase in endocytosis, consistent with the results obtained for LDL uptake in Fig. 5. *Right*, quantification of AM dye uptake for all conditions compared to DMSO. Statistics in are shown in Table S2. Scale bars, 5 μm.

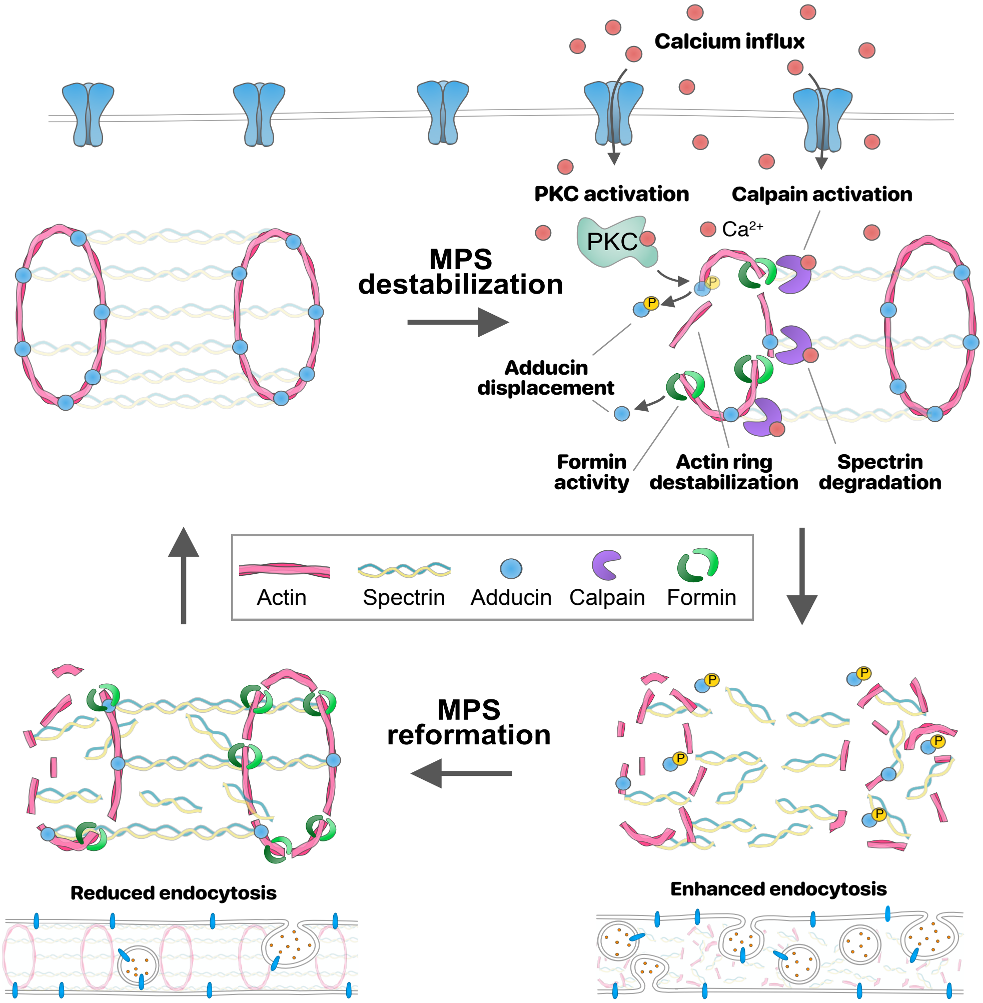

Fig. S16. Mechanistic model of MPS remodeling. When local Ca^{2+} concentration increases through neuronal activity or other processes, PKC is activated and phosphorylates adducin, an actin capping protein on the actin ring. The resulting dissociation of adducin causes actin depolymerization and destabilization of the actin–spectrin complex, promoting MPS disassembly. Adducin dissociation also enhances the susceptibility of the MPS to calpain-mediated degradation of spectrin, which promotes MPS degradation. Re-assembly of the MPS at the site of MPS degradation then allows the next cycle of remodeling. In parallel, formin plays a dual role in MPS remodeling and stability. In the short term, formin binding to actin filaments can displace adducin, promoting MPS remodeling. Formin-mediated growth of linear actin filaments is important for the reformation of MPS after disassembly, promoting long-term MPS integrity. Functionally, MPS dynamics promotes endocytosis in axons.

Supplementary Tables

	Concentration	Time	Source
**Endocytosis inhibitors**			
Pitstop2	20 μM	30 min	Sigma, SML1169
Dynasore	80 μM	30 min	Sigma, D7693
**Cytoskeletal inhibitors or other drugs**			
Nocodazole	10 μg/mL	30 min—1 h	Sigma, SML1665
Jasplakinolide	10 μM	30 min	Tocris, 2792
SMIFH2	10 or 30 μM	10 min, 1 h, or 3 h, as indicated	Sigma, S4826
CK666	50 μM	30 min	Sigma, SML0006
Blebbistatin	10 μM	30 min	Sigma, B0560
**PKC activators and inhibitors**			
GF 109203X	10 μM	30 min	Tocris, 741
Phorbol 12-myristate 13-acetate (PMA)	1 μM	30 min	Cell Signalling Technologies, 12808S
Phorbol 12,13-dibutyrate (PdBu)	2 μM	30 min	ThermoFisher, 12-011 / 1201
**CaM and CamKII inhibitors**			
W-7	50 μM	30 min	Santa Cruz, sc-201501
KN93	10 μM	30 min	Selleck Chem, S6787
KN62	10 μM	30 min	Selleck Chem, S7422
**Calpain inhibitors**			
MDL 28170	50 μM	30 min–1 h	Santa Cruz, sc-201301
Calpain inhibitor II	10 μM	30 min	Sigma, A6060
Calpastatin peptide Ac 184-210	50 μM	30 min	Sigma, SCP0063
**Calcium signaling**			
BAPTA-AM	10 μM	30 min	Sigma, A1076
NP-EGTA, AM	5–30 μM	30 min	Setareh Biotech, 6638
Thapsigargin	1 μM	30 min	Tocris, 1138
2-APB	50 μM	30 min	Tocris, 1224
**Neuronal activity**			
Tetrodotoxin (TTX)	1 μM	30 min	Biotium, 00061
**Calcium channel inhibitors**			
ω-agatoxin IVA (P/Q-type Ca^{2+} channel inhibitor	400 nM	15 min	Bio-techne, 2799
ω-conotoxin (N-type inhibitor)	1 μM	15 min	Bio-techne, 1085
Nimodipine (L-type inhibitor)	50 μM	15 min	Santa Cruz, sc-201464

Table S1. List of pharmacological inhibitors and activators. All pharmacological inhibitors and activators used in this study, along with concentrations, treatment durations, and source are indicated.

	p values	Statistical test	Replicate number (*N*)
**Figure 1**			
**Fig. 1F**, fraction of axons remodeling per field-of-view.	N/A	N/A	*N* = 185 axons from 49 FOVs (pooled from 8 independent experiments)
**Fig. 1F**, fraction axon length remodeling	N/A	N/A	*N* = 19 axons (pooled from 3 independent experiments)
**Figure 2**			
**Fig. 2B**, autocorrelation amplitude relative to DMSO control	Dynasore, 0.148<br>PitStop, 0.682<br>Nocodazole (Noco), 0.908<br>Dynarrestin, 0.313<br>Blebbistatin, 0.228<br>ROCK inhibitor (Y27632), 0.189<br>BAPTA, 0.00689<br>Ca^{2+} uncaging, 0.0384<br>Jasplakinolide (Jasp), 5.60×10^{-4}	Unpaired t-test	*N* > 20 axons (pooled from 2–3 independent experiments)
**Fig. 2C**, prevalence of remodeling, BAPTA vs. DMSO	1.65×10^{-4}	ANOVA + Tukey's HSD (DMSO control shared with Fig. 2D–E).	*N* = 10–13 axons (pooled from 2 independent experiments)
**Fig. 2D**, prevalence of remodeling, Ca^{2+} uncaging vs. DMSO	0.00403	ANOVA + Tukey's HSD (as in Fig. 2C)	*N* = 10–13 axons (pooled from 2 independent experiments)
**Fig. 2E**, prevalence of remodeling, TTX vs. DMSO	0.0186	ANOVA + Tukey's HSD (as in Fig. 2C–D)	*N* = 10–13 axons (pooled from 2 independent experiments)
**Fig. 2F**, autocorrelation amplitude relative to DMSO control	ω-agatoxin, 0.253<br>ω-conotoxin, 0.00936<br>Nimodipine, 0.00284<br>thapsigargin, 0.997<br>2-APB, 0.998	ANOVA + Tukey's HSD	*N* > 20 axons (pooled from 2–3 independent experiments)
**Figure 3**			
**Fig. 3B**, autocorrelation amplitude relative to DMSO control	W-7, 0.525<br>KN93, 0.999<br>KN62, 0.337<br>GFX, 3.5×10^{-6}<br>Calpain inhibitor II, 0.0060<br>calpastatin, 0.0192<br>MDL, 0.0412	ANOVA + Tukey's HSD	*N* > 20 axons (pooled from 2 independent experiments, CaM and CamKII inhibitors; 3 experiments, calpain inhibitors)
**Fig. 3C**, prevalence of remodeling	GFX, 0.00465<br>PMA, 0.00888	ANOVA + Tukey's HSD	*N* = 10–12 axons (pooled from 2 independent experiments)
**Fig. 3F**, normalized pAdd intensity	$<5 \times 10^{-7}$	ANOVA + Tukey's HSD	*N* > 50 axons from 5 FOVs (representative dataset of 3 experiments)
**Fig. 3G**, prevalence of remodeling	Adducin KD, 0.00891<br>GFX, 0.00732<br>GFX + adducin KD, 0.972<br>GFX vs. GFX + adducin KD, 0.0411	ANOVA + Tukey's HSD	*N* = 10–17 axons (pooled from 2 independent experiments)

Table S2. Complete list of statistical information.

	p values	Statistical test	Replicate number (*N*)
**Fig. 3H**, prevalence of remodeling, Jasp vs. DMSO	6.83×10^{-5}	ANOVA + Tukey's HSD (DMSO control shared with Fig. 4A and 4G)	$N = 12–30$ axons (pooled from 3 independent experiments)
**Fig. 3I**, prevalence of remodeling, SMIFH2 vs. DMSO	0.0275	Unpaired t-test	$N = 10$ axons (pooled from 2 independent experiments)
**Figure 4**			
**Fig. 4A**, prevalence of remodeling, MDL vs. DMSO	0.0310	ANOVA + Tukey's HSD (DMSO control shared with Fig. 3H)	$N = 12–30$ axons (pooled from 3 independent experiments)
**Fig. 4B**, spectrin intensity after Ca^{2+} uncaging	$<1 \times 10^{-7}$	ANOVA + Tukey's HSD	$N = 6$ axons (representative dataset of 3 experiments)
**Fig. 4C**, normalized cleaved spectrin levels	$<1 \times 10^{-6}$	ANOVA + Tukey's HSD	$N > 130$ axons from 10 FOVs (pooled from 2–5 independent experiments)
**Fig. 4D**, correlation of cleaved spectrin intensity with MPS degradation	2.68×10^{-5}	F-test	$N = 19$ axons from 10–20 regions (representative dataset of 3 experiments)
**Fig. 4E**, normalized cleaved spectrin levels	$<1 \times 10^{-7}$	ANOVA + Tukey's HSD	$N > 150$ axons from 5 FOVs (representative dataset of 6 experiments)
**Fig. 4F**, spectrin intensity after Ca^{2+} uncaging	GFX alone vs. GFX with Ca^{2+} uncaging, 0.0836<br>Ca^{2+} uncaging vs. no caged Ca^{2+} without GFX, $<1 \times 10^{-7}$	ANOVA + Tukey's HSD	$N = 5$ axons (representative dataset of 2 experiments)
**Fig. 4G**, prevalence of remodeling	Jasp, 6.83×10^{-5}<br>MDL, 0.0310<br>Jasp + MDL, 8.60×10^{-6}<br>Jasp vs. MDL, 0.0125<br>Jasp vs. Jasp + MDL, 0.906<br>MDL vs. Jasp + MDL, 0.0011	ANOVA + Tukey's HSD (DMSO control shared with Fig. 3H)	$N = 12–30$ axons (pooled from 3 independent experiments)
**Figure 5**			
**Fig. 5B**, LDL uptake	Dynasore, GFX, MDL, Jasp, $<1 \times 10^{-7}$<br>βII-spectrin KD, 0.00709	ANOVA + Tukey's HSD	$N = 30–60$ axons pooled from 5 FOVs (representative dataset of 2 experiments)
**Figure S1**			
**Fig. S1C**, prevalence of remodeling	N/A	N/A	$N = 8$ axons (pooled from 2 independent experiments)
**Fig. S1F**, prevalence of remodeling	N/A	N/A	$N = 17$ axons (pooled from 2 independent experiments)
**Figure S2**			

Table S2. Complete list of statistical information (continued).

	***p* values**	**Statistical test**	**Replicate number (*N*)**
**Fig. S2B**, fluorescence intensity before vs. after disassembly	Spectrin, 5.87×10^{-6}<br>CellBrite, 0.617	Unpaired t-test	*N* = 10 transitions (pooled from 2 independent experiments)
**Fig. S2B**, fluorescence intensity before vs. after reassembly	Spectrin, 1.05×10^{-5}<br>CellBrite, 0.610	Unpaired t-test	*N* = 13 transitions (pooled from 2 independent experiments)
**Fig. S2C**, correlation of fluorescence intensity	N/A	N/A	*N* = 20 regions from 4–5 axons; 240 total transitions (pooled from 2 independent experiments)
**Figure S4**			
**Fig. S4A**, prevalence of remodeling, proximal vs. distal	$<1 \times 10^{-7}$	Unpaired t-test	*N* = 19 medial–distal axons, 10 proximal axons (pooled from 4 independent experiments, medial–distal axons; 2 experiments, proximal axons)
**Fig. S4C**, prevalence of remodeling, synaptic vs. non-synaptic	0.2948	Unpaired t-test	*N* = 10–20 synaptic and non-synaptic sites from 5 axons (pooled from 2 independent experiments)
**Fig. S4D**, number of remodeling regions in synaptic vs. non-synaptic sites	0.0142	Unpaired t-test	*N* = 20–60 remodeling regions per axon from 5 axons (same dataset as in Fig. S4C; pooled from 2 independent experiments)
**Figure S5**			
**Fig. S5C**, percent axons with intact MPS	N/A	N/A	*N* = 66 axons from 27 FOVs (pooled from 3 independent experiments)
**Figure S6**			
**Fig. S6C**, autocorrelation amplitude	Spectrin-mNeonGreen vs. CellBrite, 0.0222<br>Spectrin-mNeonGreen vs. cytoplasmic GFP, 0.00642<br>CellBrite vs. cytoplasmic GFP, 0.965	ANOVA + Tukey's HSD	*N* = 8–14 axons (representative dataset of 2 experiments)
**Figure S7**			
**Fig. S7B**, resting calcium levels compared to DMSO control	BAPTA, $<1 \times 10^{-7}$<br>TTX, 0.991<br>Nimodipine, 0.992<br>ω-conotoxin, 0.880<br>nimodipine and ω-conotoxin, 0.0425	ANOVA + Tukey's HSD	*N* = 50–80 axons per condition from 5 FOVs (pooled from 2 independent experiments)
**Figure S9**			
Autocorrelation amplitude	GFX, 0.00146<br>PMA, 5×10^{-7}	ANOVA + Tukey's HSD	*N* = 28–50 axons per condition from 5 FOVs (pooled from 2 independent experiments)
**Figure S10**			

Table S2. Complete list of statistical information (continued).

	p values	Statistical test	Replicate number (*N*)
**Fig. S10C**, MPS integrity (autocorrelation amplitude compared to scramble shRNA)	Spectrin, 0.00950<br>Adducin, 0.0345	Unpaired t-test	*N* = 30–40 axonal regions from 3 FOVs (pooled from 3 independent experiments)
**Figure S11**			
**Fig. S11A**, autocorrelation amplitude compared to DMSO	SMIFH2, 0.0238<br>CK666, 0.486	ANOVA + Tukey's HSD	*N* = 15–20 axons from 5 FOVs (pooled from 5 independent experiments, SMIFH2; 1 experiment, CK666)
**Figure S12**			
**Fig. S12B**, Spatial autocorrelation amplitude, spectrin	1 h vs. 0 h, 0.340<br>3 h vs. 0 h, 0.000764<br>3 h vs. 1 h, 0.0304	ANOVA + Tukey's HSD	*N* = 60–80 regions from 3 FOVs (representative dataset of 3 experiments)
**Fig. S12B**, Spatial autocorrelation amplitude, actin	1 h vs. 0 h, 0.0160<br>3 h vs. 0 h, 0.00432<br>3 h vs. 1 h, 0.374	ANOVA + Tukey's HSD	*N* = 60–80 regions from 3 FOVs (representative dataset of 3 experiments)
**Fig. S12C**, Spatial autocorrelation amplitude	SMIFH2 1 h vs. 0 h, 0.0535<br>SMIFH2 3 h vs. 0 h, 0.0153<br>SMIFH2 vs. Bleb, 1 h, 3.13×10^{-5}<br>SMIFH2 vs. Bleb, 3 h, 0.00205	ANOVA (two-way) + Tukey's HSD	*N* = 60–100 regions from 5 FOVs (pooled from 2 independent experiments)
**Fig. S12D**, Spatial autocorrelation amplitude (SMIFH2 vs. DMSO control)	1 h, 0.810<br>3 h, 0.089	ANOVA + Tukey's HSD	*N* = 30–50 regions from 5 FOVs (pooled from 2 independent experiments)
**Figure S13**			
**Fig. S13B**, fluorescence intensity	3.90×10^{-8}	Unpaired t-test	*N* = 31 mDia KD axons and 70 wildtype (WT) axons (pooled from 2 independent experiments)
**Fig. S13C**, Spatial autocorrelation amplitude	1.17×10^{-5}	Unpaired t-test	*N* = 24 KD axons and 70 uninfected control axons from 5 FOVs (pooled from 2 independent experiments)
**Figure S14**			
**Fig. S14B**, Spatial autocorrelation amplitude	0.0021	Unpaired t-test	*N* = 20–25 regions per condition from 6–7 FOVs (representative dataset of 2 experiments)
**Figure S15**			
**Fig. S15A**, endocytosis of DMSO vs. dynasore	4.68×10^{-8}	Unpaired t-test	*N* > 60 axons from 5 FOVs (representative dataset of 2 experiments)
**Fig. S15B**, AM dye uptake	$<1 \times 10^{-7}$	ANOVA + Tukey's HSD	*N* = 100–150 axons from 10 FOVs (representative dataset of 2 experiments)

Table S2. Complete list of statistical information (continued).

Movie S1 caption

Movie S1. Live-cell Lattice SIM imaging of the MPS in DIV15 spectrin mNeonGreen neurons at 15-sec resolution, revealing stereotypical disassembly and reformation dynamics of the MPS in regions along the axon.
